# tRNA epitranscriptome determines pathogenicity of the opportunistic pathogen *Pseudomonas aeruginosa*

**DOI:** 10.1073/pnas.2312874121

**Published:** 2024-03-07

**Authors:** Jonas Krueger, Matthias Preusse, Nicolas Oswaldo Gomez, Yannick Noah Frommeyer, Sebastian Doberenz, Anne Lorenz, Adrian Kordes, Svenja Grobe, Mathias Müsken, Daniel P. Depledge, Sarah L. Svensson, Siegfried Weiss, Volkhard Kaever, Andreas Pich, Cynthia M. Sharma, Zoya Ignatova, Susanne Häussler

**Affiliations:** ^a^Institute for Molecular Bacteriology, Center of Clinical and Experimental Infection Research (TWINCORE), a joint venture of the Hannover Medical School and the Helmholtz Center for Infection Research, Hannover 30625, Germany; ^b^Research Core Unit Proteomics and Institute for Toxicology, Hannover Medical School, Hannover 30625, Germany; ^c^Department of Molecular Bacteriology, Helmholtz Center for Infection Research, 38124 Braunschweig, Germany; ^d^Cluster of Excellence “Resolving Infection susceptibility” (RESIST), Hannover Medical School, Hannover 30625, Germany; ^e^Research Core Unit Metabolomics and Institute of Pharmacology, Hannover Medical School, Hannover 30625, Germany; ^f^Central Facility for Microscopy, Helmholtz Centre for Infection Research, Braunschweig 38124, Germany; ^g^Institute of Virology, Hannover Medical School, Hannover 30625, Germany; ^h^German Center for Infection Research, Partner Site Hannover-Braunschweig, Hannover 30625, Germany; ^i^Department of Molecular Infection Biology II, Institute of Molecular Infection Biology, University of Würzburg, Würzburg 97080, Germany; ^j^Institute of Immunology, Medical School Hannover, Hannover 30625, Germany; ^k^Institute for Biochemistry and Molecular Biology, University Hamburg, 20146, Germany; ^l^Department of Clinical Microbiology, Copenhagen University Hospital—Rigshospitalet, Copenhagen 2100, Denmark

**Keywords:** translation, riboseq, tRNA modification, epitranscriptomics, pathogenicity

## Abstract

The success of bacterial pathogens depends on the coordinated expression of virulence determinants. Regulatory circuits that drive pathogenesis are complex, multilayered, and incompletely understood. Here, we reveal that alterations in tRNA modifications define pathogenic phenotypes in the opportunistic pathogen *Pseudomonas aeruginosa*. We demonstrate that the enzymatic activity of GidA leads to the introduction of a carboxymethylaminomethyl modification in selected tRNAs. Modifications at the wobble uridine base (cmnm^5^U_34_) of the anticodon drives translation of transcripts containing rare codons. Specifically, in *P. aeruginosa* the presence of GidA-dependent tRNA modifications modulates expression of genes encoding virulence regulators, leading to a cellular proteomic shift toward pathogenic and well-adapted physiological states. Our approach of profiling the consequences of chemical tRNA modifications is general in concept. It provides a paradigm of how environmentally driven tRNA modifications govern gene expression programs and regulate phenotypic outcomes responsible for bacterial adaption to challenging habitats prevailing in the host niche.

## Significance

*Pseudomonas aeruginosa* is an opportunistic pathogen, renowned for its ability to adapt to challenging conditions. In this study, we elucidated how the activity of a single gene orchestrates pathogenicity. To achieve this, we integrated systems-level transcriptomic, ribosome profiling, and proteomic data, along with virulence datasets from over 400 clinical isolates. Our investigation focuses on the post-transcriptional consequences of GidA-dependent carboxymethylaminomethyl modifications in specific transfer RNAs (tRNAs). Through this research, we demonstrate how alterations in tRNA modifications exert control over gene expression programs. Consequently, we shed light on mechanistic insights into how bacteria govern cellular proteomic shifts, leading to pathogenic and well-adapted physiological states. This finding opens up exciting opportunities for developing pathoblockers to combat life-threatening diseases caused by highly problematic pathogens.

Comparative genomic approaches have enabled the identification of previously unknown determinants of bacterial pathogenicity and resulted in an impressive amount of data on candidate virulence genes (1, 2). However, even within single bacterial species, the pathogenic potential of different strains may vary substantially despite the fact that they share most, if not all genes (3). Likewise, it has been demonstrated for *Pseudomonas aeruginosa* that genes essential for pathogenicity of one strain are neither required nor predictive for the pathogenicity of others. Virulence of this important opportunistic pathogen is both multifactorial and combinatorial (4). A pool of pathogenicity-associated genes interacts in various combinations in different environments and genetic backgrounds, complicating the development of anti-virulence drugs as new options to combat problematic, often multi-drug resistant, pathogens.

A considerable diversity of mechanisms is found among bacteria to regulate expression of virulence genes in response to conditions prevailing in the host (5–7). Numerous conserved and species-specific regulatory proteins have been identified, including global transcriptional regulators of virulence networks such as those involved in inter-bacterial communication (quorum sensing) (8–11). In addition, advances in high-throughput sequencing have facilitated the identification of regulatory non-coding RNAs that added multiple layers of post-transcriptional control on virulence-related pathways (12–14). Furthermore, it has recently been discovered that tRNA modifications play important roles in the regulation of bacterial pathogenicity (15–18).

The decoding properties of tRNAs are influenced by posttranscriptional modifications particularly of the anticodon (at the wobble base position 34) and/or critical positions (e.g., position 37) of the anticodon-stem loop (19, 20). As most amino acids are encoded by two or up to six codons, codon choice can affect decoding specificity and thus modulate translation efficiency of selected genes (21). tRNA modifications have key roles in stress responses (22–24) Lack of modifications leads to complex pathologies in eukaryotes (25–33) and affect expression of virulence genes in prokaryotes (34–38). A prominent example of a bacterial tRNA modifying enzyme is GidA (MnmG), which acts in concert with MmnE to modify the anticodon wobble position in selected tRNAs of *Escherichia coli* (39). In *P. aeruginosa*, GidA was demonstrated to be involved in the regulation a major quorum sensing regulator (RhlR) (37, 40, 41), and more recently, it has been shown that GidA-dependent tRNA modification is crucial for oxidative stress response and biofilm formation (42). However, the influence of tRNA modifications on the regulatory circuits of pathogenicity mechanisms has not been fully elucidated for a wide array of bacterial infectious diseases.

In the present work, we quantified GidA-dependent tRNA modifications in total and purified tRNAs and integrated transcriptomic, ribosome profiling, and proteomic data to demonstrate the post-transcriptional consequences of the absence of carboxymethylaminomethyl (cnmn) modifications in selected tRNAs on the virulence of *P. aeruginosa*. Our results show that modulation of tRNA modification adds another layer of regulation in the transfer of information from DNA to protein and determines very complex phenotypes, such as bacterial pathogenicity.

## Results

### GidA Function in Host–Pathogen Interaction.

**Fig. 1.**
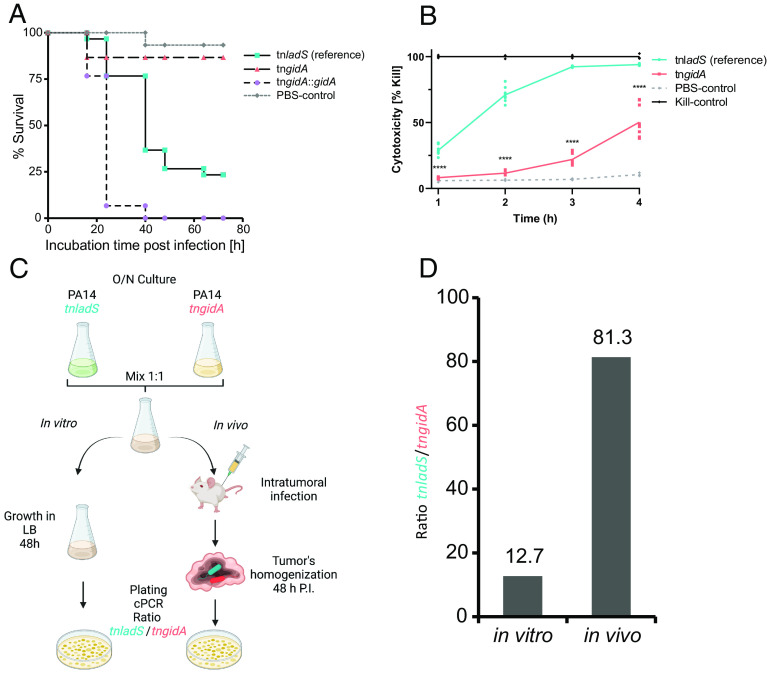
GidA is essential for *P. aeruginosa* fitness and virulence in vivo and in vitro. (*A*), *Galleria mellonella* survival assay. Ten *G. mellonella* larvae per replicate were infected with 100 bacteria of the PA14 tn*gidA* and the tn*ladS* reference harboring the empty vector, and the tn*gidA* mutant complemented with *gidA* on the pUCP20 vector *in trans* (tn*gidA*::*gidA*). PBS served as a control. Dead larvae were counted at indicated time points following incubation of the larvae at 37 °C. Shown are mean values from triplicates. (*B*) Experimental design of the in vivo competition assay. A 1:1 mixture of overnight (O/N) grown tn*ladS* reference and tn*gidA* was used to inoculate LB medium (in vitro condition) and to infect BALB/c mice harboring a subcutaneous CT26 tumor (in vivo condition). Dilutions of the mixed LB-culture or homogenized CT26 tumors were plated on LB agar plates and colonies were subjected to colony PCR. (*C*) Ratios of tn*ladS*/tn*gidA* mutant colonies were calculated for in vivo (n = 9) and in vitro (n = 5) conditions. (*D*), Cytotoxicity of *P. aeruginosa* on RAW 264.7 cells. RAW cells were infected at a MOI of 1 for 4 h. PBS served as a negative control and 10% (v/v) Triton-X100 as killing control. Cell viability at each time point was determined by measuring the LDH activity in the supernatant, and expressed as a percentage of the activity in the killing control. Mean ± SD is displayed. ****P < 0.0001 in one-way ANOVA, with the post hoc Dunnett test.

To study the function of GidA in *P. aeruginosa* pathogenicity, we employed a PA14 *gidA* transposon mutant (PA14 *gidA*::MrT7, tn*gidA*) (43) and characterized its virulence-associated phenotypes in comparison to a reference strain bearing the same transposon, inserted in a non-functional gene (PA14 *ladS*::MrT7, tn*ladS*) (44). First, we tested the pathogenic potential of the two strains by infecting larvae of the wax moth *Galleria mellonella.* The defense system of *G. mellonella* larvae shows functional similarities to the human innate immune system and has therefore been increasingly used as a surrogate to study host–pathogen interactions with a range of microorganisms (45–47). We found that the wild-type (WT) like reference strain tn*ladS* killed 75% of the larvae within 48 h. In contrast, tn*gidA* was almost avirulent. Complementing *gidA* (tn*gidA*::*gidA*) in *trans* under control of a constitutive promoter led to increased mortality rates. More than 90% of the larvae were dead after 24 h (Fig. 1*A*), suggesting that GidA is essentially involved in the pathogenicity of *P. aeruginosa*.

To confirm this finding in an in vivo system that also involves an adaptive immune system, we tested the tn*gidA* mutant in our established mouse tumor model (48). In this model, intravenously injected *P. aeruginosa* colonize solid tumors and form biofilms within the cancerous tissue. In this host niche, *P. aeruginosa* triggers a transcriptional response similar to that of *P. aeruginosa* cells in cystic fibrosis lungs (49). In pilot experiments, we observed that the tn*gidA* and tn*ladS* strain showed no difference in their ability to colonize mouse tumors. Thus, we proceeded with competition experiments. Tumor-bearing mice were infected with a 1:1 mixture of the tn*gidA* and tn*ladS* strains (Fig. 1*B*). A competitive disadvantage of the tn*gidA* mutant was already apparent in the in vitro LB co-culture, with over 10-fold difference in the recovery of the tn*gidA* mutant versus the tn*ladS* strain. However, in vivo the effect was much more pronounced, with 80-fold more tn*ladS* colonies recovered after infection compared to tn*gidA* (Fig. 1*C*). In an in vitro model using a murine macrophage cell line (RAW 264.7), we also observed that tn*gidA* was significantly less cytotoxic than the reference strain (Fig. 1*D*). Thus, inactivation of *gidA* severely interferes with the virulence and in vivo fitness of *P. aeruginosa*.

**Fig. 2.**
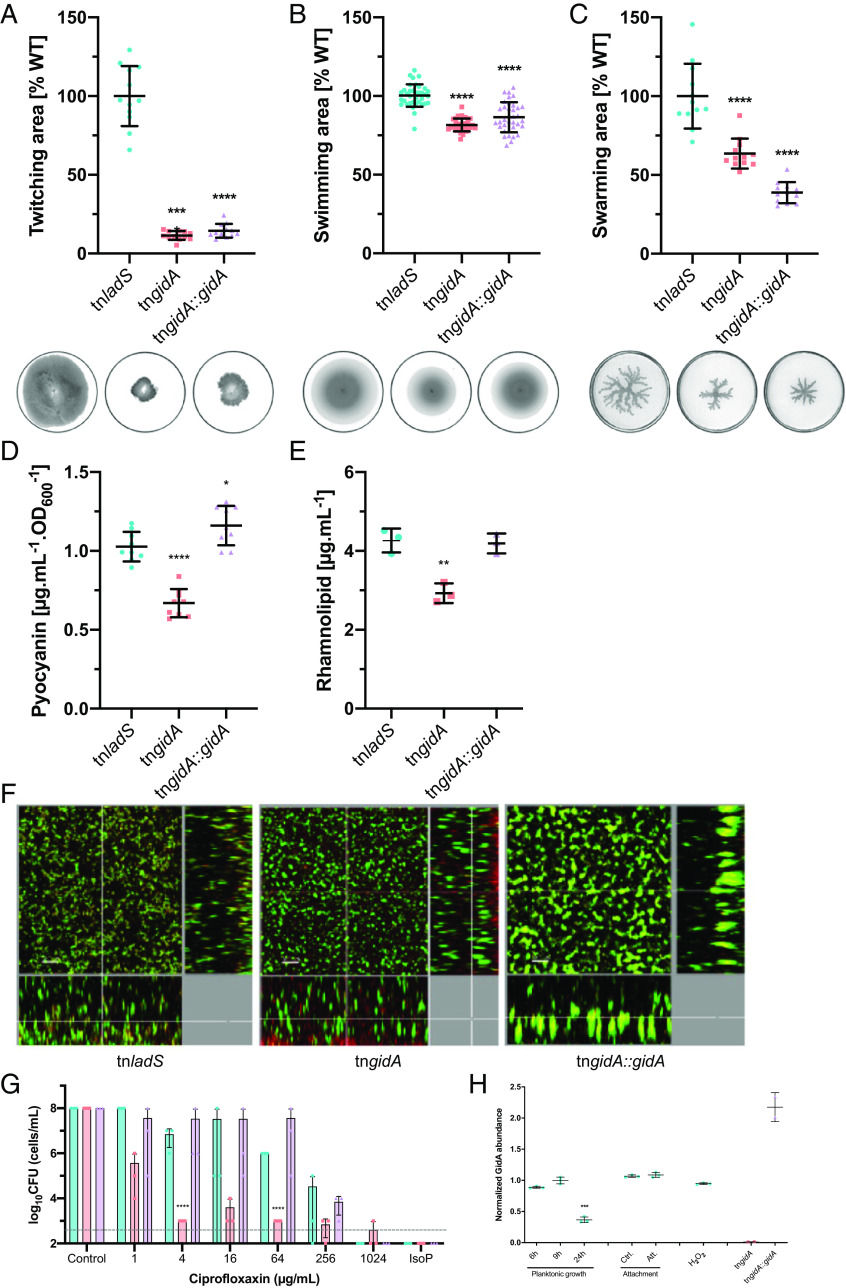
Phenotypic characterization of the PA14 tn*gidA* mutant. Motility of the tn*gidA* (*■*), tn*ladS* (●), and complemented tn*gidA* (▲). (*A*) Twitching area measured after 48 h (n = 16, from three independent biological replicates). (*B*) Swimming area measured after 48 h on 0.3% agar BM2 medium (n = 36, from three independent biological replicates). (*C*) Swarming area measured after 24 h on 0.5% agar BM2 medium (n = 16, from three independent biological replicates). Images are representative of one replicate from each experiment. Mean ± SD is displayed. *P < 0.05, **P < 0.01, ***P < 0.001, ****P < 0.0001 in one-way ANOVA (with the post hoc Dunnett test). (*D*) Pyocyanin and (*E*) rhamnolipid concentration normalized to OD_600_ were measured. (*F*) Biofilm images were taken after 48 h incubation by confocal microscopy using the LIVE/DEAD staining kit (BacLight Bacterial Viability kit, Molecular Probes/Invitrogen). Living (green) and dead (red) cells are visualized using the IMARIS software package (version 5.7.2, Bitplane). The central image shows the top view; *Lower* and *Right* images display the side view of the biofilm. Representative images (image section of 290.63 µm × 290.63 µm) for each strain are depicted. (*G*) Biofilms were grown for 24 h in microtiter plates and subjected for additional 24 h to ciprofloxacin at the indicated concentrations. The responsiveness toward the antibiotic was monitored by determining the CFU. Ctrl: control without antibiotic treatment; IsoP: isopropanol treatment; dotted gray line corresponds to the detection limit (CFU ≤ 100 cells/mL). Data are mean ± SD (n = 3). ****P < 0.0001 in two-way ANOVA (with post-hoc test Tukey). (*H*) GidA protein expression was determined by targeted LC–MS/MS based single reaction monitoring (SRM) analysis following growth of PA14 tn*ladS* cells under different culture conditions. The tn*gidA* mutant and the *gidA* overexpressing strain (PA14 tn*gidA*::*gidA*) were grown in LB for 7.5 h. Data are mean (horizontal line) ± SD (n = 3 for conditions and n = 2 for control samples). * indicates significantly altered values with a *P*-value ≤ 0.05 and *** indicates *P*-value ≤ 0.001 as determined by Student’s *t* test.

In vitro, the growth of tn*gidA* was slightly affected (*SI Appendix*, Fig. S1*A*), corroborating earlier observations (37). In contrast, at late growth phase, the tn*gidA* mutant displayed higher OD_600_ values (*SI Appendix*, Fig. S1*A*) and produced significantly more colony-forming units (CFU) than the reference strain (*SI Appendix*, Fig. S1*B*). The tn*gidA* mutant was also affected in motility, namely twitching, swimming, and swarming (Fig. 2 *A*–*C*) Overexpression of *gidA in trans* did not fully restore motility. Interestingly, it was observed earlier that both ablation and forced overproduction of the tRNA-modifying enzyme MiaA induced proteome changes in *E. coli* due to stimulated translational frameshifting (50). The two RhlR-regulated virulence factors, pyocyanin and rhamnolipid, were produced at lower amounts in the tn*gidA* mutant (Fig. 2 *D* and *E*). The latter effect was expected, as GidA is known to impact the expression of RhlR, which upon binding of its cognate autoinduced butanoyl-homoserine lactone (C_4_-HSL), controls expression of quorum sensing genes (37).

Confocal microscopy imaging of live/dead stained biofilms showed that disruption of *gidA* altered the structure of *P. aeruginosa* biofilms, resulting in more dead bacteria at the bottom of the biofilm (Fig. 2*F*). More importantly, the quinolone antibiotic ciprofloxacin was markedly more effective against tn*gidA* biofilms compared to the control or the complemented strain (Fig. 2*G*).

Finally, we determined the protein expression level of GidA under various culture conditions (Fig. 2*H*). Expression levels were stable during exponential and early stationary growth phases, while they significantly decreased at late stationary phase. No significant GidA protein level changes were observed under various types of environmental stress. Thus, GidA protein levels are subject to a dynamic expression throughout the growth phases.

### Identification of *P. aeruginosa* GidA-Dependent tRNA Modifications by Multiple Reaction Monitoring.

**Fig. 3.**
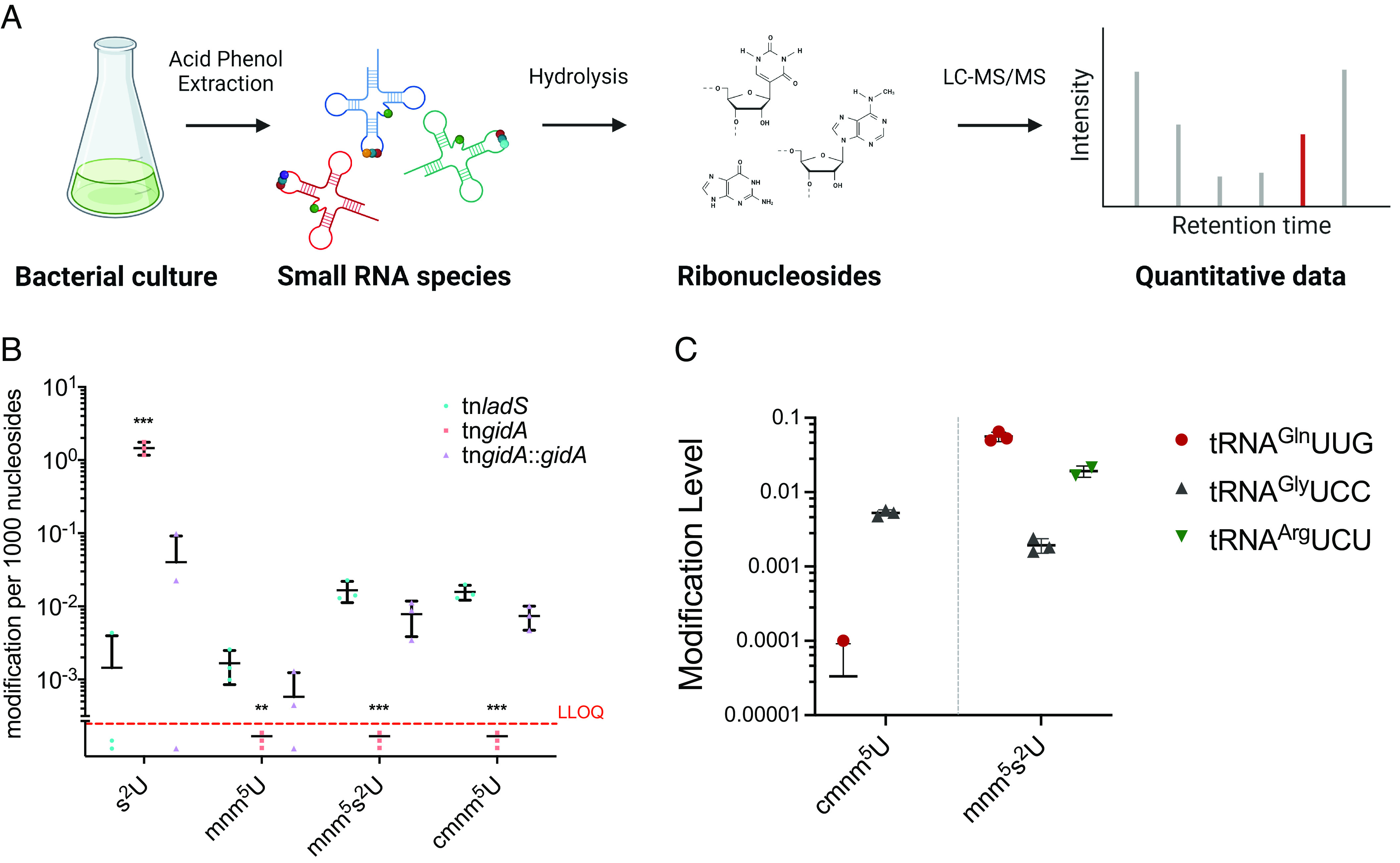
Targeted LC–MS/MS based quantification of modified tRNA uridine derivatives and characterization of their impact on translation efficiency. (*A*) Schematic overview of the workflow for LC–MS/MS-based quantification of modified uridines. (*B*) The quantities of modified ribonucleosides shown as modification per 1,000 nucleosides were quantified for the PA14 tn*gidA* (■) and the tn*ladS* (●) (both harboring the pUCP20 empty vector), and the tn*gidA* complemented (tn*gidA*::*gidA*,▲). s^2^U = 2-thiouridine, mnm^5^U = 5-methylaminomethyluridine, mnm^5^s^2^U = 5-methylaminomethyl-2-thiouridine, cmnm^5^U = 5-carboxymethylaminomethyluridine. The dotted red line corresponds to a value < lower limit of quantification (LLOQ). (*C*) Quantities of GidA-dependent modifications in tRNA^Gln^UUG, tRNA^Gly^UCC, and tRNA^Arg^UCU from PA14 tn*ladS*. The quantities of modified ribonucleosides for each individual tRNA were analyzed by MRM external standard calibration. The modification level (modified ribonucleoside (fmol] × number of parent ribonucleoside in tRNA sequence/parent ribonucleoside [fmol] + detected modified ribonucleosides that descends from the respective parent nucleosides [fmol]) was determined according to Grobe et al. (53).

In *E. coli*, the GidA homologue targets six tRNAs harboring a uridine at the wobble position (U_34_) of the anticodon: tRNA^Lys^UUU, tRNA^Glu^UUC tRNA^Gln^UUG, tRNA^Leu^UAA tRNA^Arg^UCU, and tRNA^Gly^UCC (51). We therefore investigated GidA-dependent alterations of tRNA modifications in *P. aeruginosa* using a targeted LC–MS-based multiple reaction monitoring (MRM) approach. The total RNA fraction was isolated from tn*gidA* and tn*ladS*, enriched for small RNAs and digested to single nucleotides. The relative quantities of modified uridines were then quantified using chemically synthesized modified uridines as a reference (Fig. 3*A* and *SI Appendix*, Table S1). This confirmed that GidA is required for addition of the cmnm modifications to uridine, since inactivation of *gidA* resulted in a marked loss of cmnm^5^U and the related modifications mnm^5^U and mnm^5^s^2^U (Fig. 3*B*). Of note, modified uridine s^2^U was significantly increased in the *gidA*-deficient mutant (Fig. 3*B*), as previously observed for *E. coli* and *P. aeruginosa* (42, 52). Expression of *gidA* in *trans* in *P. aeruginosa* tn*gidA* restored cmnm^5^U residues almost to levels of the reference strain (Fig. 3*B*).

We next measured the level of GidA-dependent cmnm modifications of uridines in single tRNAs. We purified three individual tRNAs (tRNA^Gln^UUG, tRNA^Arg^UCU, and tRNA^Gly^UCC) from the small RNA-enriched fraction of total RNA using complementary DNA oligonucleotides attached to magnetic beads. GidA-dependent modifications of purified tRNAs were quantified using a LC–MS-based MRM approach with external standard calibration and synthetic standard as references. In all three tRNAs, cmnm modification of uridines was detected following their isolation from the PA14 tn*ladS*. Thereby, the GidA-dependent wobble modification mnm^5^s^2^U was identified as the final derivative in tRNA^Gln^UUG and tRNA^Arg^UCU, whereas, in tRNA^Gly^UCC, cmnm^5^U was the dominant derivative in addition to low levels of mnm^5^s^2^U (Fig. 3*C*). We could also show that the GidA-dependent cmnm^5^U, mnm^5^s^2^U and the related s^2^U derivatives were absent in tRNA^Gln^UUG and tRNA^Gly^UCC isolated from tn*gidA,* while the level of the other modifications was the same as compared to tn*ladS* (*SI Appendix*, Fig. S2 *A* and *B*).

### GidA Modulates Translation Efficiency at Selected Codons.

**Fig. 4.**
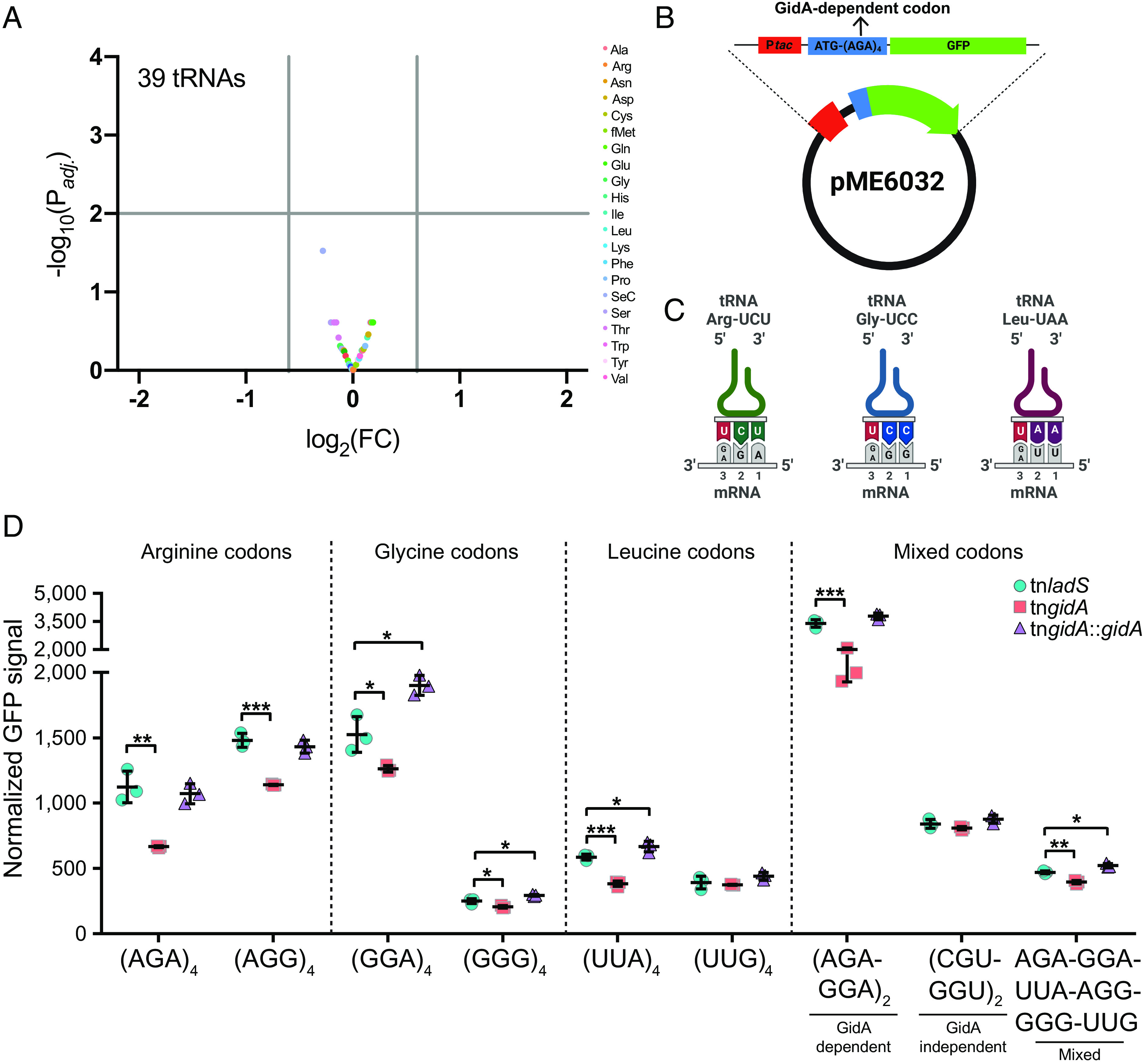
GidA modulates translation efficiency at selected codons. (*A*) Volcano plot depicting tRNA abundance in the tn*gidA* strain compared to the control tn*ladS* strain. We determined significance as an adjusted log10 *P* < 0.01 and an absolute log2-fold change greater than ±0.6 (gray lines on the *x* and *y* axes, respectively). (*B*) Schematic overview of GFP-based reporter on the IPTG-inducible pME6032 vector with AGA as exemplary GidA*-*dependent codon. The reporters were introduced in *trans* into PA14 tn*gidA*, the tn*ladS* reference strain and the complemented tn*gidA* mutant harboring *gidA* on the pUCP20 plasmid. GFP expression was normalized to the OD_600_ value. (*C*) Schematic overview of the analyzed codons with the corresponding tRNA isoacceptor and the anticodon-codon binding with either on a Watson–Crick interaction (A-U) or a wobble interaction (G-U). (*D*) Translation efficiency of four consecutive arginine, glycine, or leucine codons as well as a selection of mixed codons are depicted. Mean ± SD of three biological replicates are shown, *P < 0.05, **P < 0.01, ***P < 0.001, ****P < 0.0001, one-way ANOVA (with the post hoc Dunnett test).

Based on work in *E. coli* (51) and our finding that GidA is responsible for the posttranscriptional modification of uridines at the wobble U_34_ position of selected tRNAs, we hypothesized that the decoding properties of the respective tRNAs would be affected in the absence of these modifications. To test this hypothesis, we first ensured that the lack of modifications does not affect the tRNA abundance (e.g., because of a changed tRNA half-life). To this end, we quantified the native tRNAs from our control and tn*gidA* strains using the Nano-tRNA-seq method (54). We found that our biological replicates had low variability (*SI Appendix*, Fig. S3). Notably, the tRNA levels were similar between both strains (Fig. 4*A*), suggesting that the lack of modification does not alter the abundance of tRNAs (55, 56). We then constructed reporter plasmids that encoded consecutive stretches of four arginine, glycine, or leucine codons directly after the start codon and in frame of the GFP gene (Fig. 4*B*). To explore the impact of U_34_ modifications, we used synonymous codons relying on either wobble base pairing (U-G) or a Watson and Crick (U-A) interaction at this position (Fig. 4*C*). Codons involving U-A pairing, namely Arg AGA, Gly GGA, and Leu UUA, led to a significantly lower fluorescence produced in the tn*gidA* strain (Fig. 4*D*). Complementation of the *gidA* mutation restored GFP levels or in some cases produced higher GFP signals (Fig. 2*H*). In contrast, in reporters where the U_34_ of the anticodon needs to establish a wobble interaction with guanosine, the effects were either weaker (Arg AGG, Gly GGG), or no variation of fluorescence was detected (Leu UUG). We also designed reporters harboring combinations of either different *gidA*-dependent codons (AGA, GGA) or of different *gidA*-independent codons (CGU, GGU), which showed a reduced or unchanged translation efficiency in the tn*gidA* mutant, respectively (Fig. 4*D*). Furthermore, a combination of all six codons that are decoded by tRNAs harboring a modified U_34_ in *E. coli* (e.g., the AGA-GGA-UUA-AGG-GGG-UUG stretch) revealed an overall reduced translation efficiency in the tn*gidA* mutant, albeit to a lower extent compared to stretches composed solely of the arginine AGA and leucine UUA codons (Fig. 4*D*). Together, our findings establish the GidA-dependent impact on translation efficiency of specific codons.

### Post-Transcriptional Regulatory Impact of GidA.

**Fig. 5.**
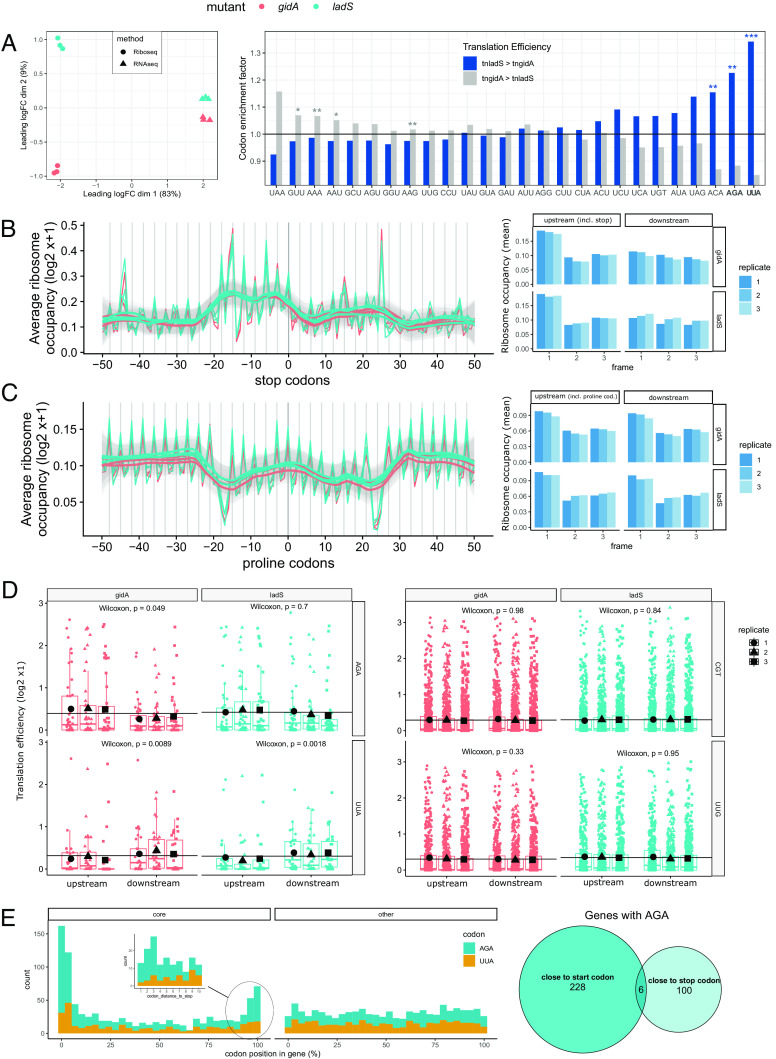
Decreased ribosome occupancy at AGA codons in *P. aeruginosa tn*gidA. (*A*) *Left*: MDS plot indicates a stronger difference between tn*gidA* and tn*ladS* in RPFs (read length 25 to 42) than in RNAseq data. *Right*: Codon enrichment (codon presence versus absence) in transcript groups with higher or lower translation efficiency (the ratio of ribosome footprints to mRNA), respectively in the tn*gidA* mutant compared to tn*ladS*. Translation efficiency was computed for each transcript in the tn*gidA* and tn*ladS* strains and the top 25% genes whose log2 fold change was higher in the tn*gidA* vs. tn*ladS* (translation efficiency tn*gidA* > tn*ladS*, in gray) and vice versa (translation efficiency tn*ladS* > tn*gidA*, in blue) were selected. The codon enrichment factor (*y* axis) of each of the two gene groups was quantified as the ratio of transcripts containing a specific codon as compared to those lacking that codon (enrichment factor). Significance was determined using hypergeometric testing. Codons with a codon enrichment score < 1.01 are not shown (*Materials and Methods*). AGA and UUA also had the strongest enrichment in an independent repetition experiment. Asterisks indicate Benjamini and Hochberg adjusted *P* values: *P ≤ 0.05, **P ≤ 0.01, ***P ≤ 0.001. (*B*) Average ribosome occupancy plot (*Left*) of genes aligned at their stop codons (UAA, UAG, UGA) using the 3′-assignment (A site) of 40 and 41 nt long reads from *P. aeruginosa*. The barplot (*Right*) illustrates the decrease in periodicity after the stop codon. For the region from −50 nt to the stop codon (including the stop codon), the reads are mainly on the first frame (mean value), which applies for all replicates (staged barplots). This effect disappears from nt 1 to 50 after the stop codon. (*C*) Same as *B* using centered proline codons (CCC, CCG, CCT, CCA). (*D*) Changes in ribosome occupancy at nt position 9, 12, 15, 18, and 21 up- and downstream of AGA, UUA, CGU, and UUG codons. Each point represents the ribosome occupancy (*y* axis, log2) of a single codon in tn*gidA* (cyan) and tn*ladS* (cyan). 1 to 3 denote different replicate with its median (horizontal red/cyan line) and mean values (black points); the black horizontal line represents the mean for each codon and mutant strain group. The *P* values were calculated comparing the region up- and downstream of each codon. (*E*) *Left*: position of AGA (blue) and UUA (orange) codons within genes (%) of *P. aeruginosa* UCBPP-PA14. UUA codons accumulated at close proximity (up to 10 codons distance) of the start codons of core genes, AGA codons—at close proximity to start and stop codons, with mainly three codons distance to stop codons (see inserted plot). *Right*: AGA codons with up to 10 codons distance from start/stop codons. The VENN diagram indicates that AGA codons are either with high proximity to the start (228 AGA codons) or stop codon (100 AGA codons) of genes only six transcripts are with both.

Since we observed that the absence of *gidA* affects the translation of AGA and UUA codons (Fig. 4*D*), we used ribosome profiling (RiboSeq) to shed light on the global effect of GidA across all translated transcripts (also named “translatome”) (57). We inferred the position of actively translating ribosomes through deep-sequencing of ribosome-protected mRNA footprints (RPF) generated by nucleolytic digestion of ribosome-mRNA complexes (58). A multidimensional scaling analysis (MDS), allowing clustering of the samples based on pairwise distances between the top 500 genes (Fig. 5 *A*, *Left*), revealed a low variability between biological replicates. Furthermore, the RiboSeq profiles were clearly different from RNASeq profiles (dimension 1, *X*-axis), which were recorded under the same culture conditions, thus highlighting differences between translational and transcriptional programs. The difference between the tn*gidA* and the tn*ladS* strains was best captured by their translatomes (dimension 2, *y*-axis).

We next computed the translation efficiency (TE, i.e., the ratio of ribosomal footprints/mRNA, also referred to as ribosome density) for each transcript in tn*gidA* and compared it to tn*ladS* by computing a codon enrichment factor (Fig. 5 *A*, *Right*). In agreement with our translational reporter approach (Fig. 4), genes containing one or more UUA or AGA exhibited an overall reduced TE. We noted that the TE of transcripts containing ACA codons also decreased. Importantly, we observed no variation of TE at isoencoders UUG and AGG codons, indicating that this effect is independent of the amino acid encoded but instead depends on the nature of the cognate tRNA. We also observed that the genes containing one or more GUU, AAA, AAU, and AAG codons showed a higher TE. The decreased (ACA) or increased (GUU, AAA, AAU, and AAG) codon-dependent variation of TE was not due to altered tRNA abundance (Fig. 4*A*) and therefore could be the result of global variations in the translational program (Fig. 5 *A*, *Left*) in the absence of GidA-dependent modifications affecting translation of UUA and AGA codons.

To explore the impact of tRNA modifications on translation in the tn*gidA* mutant further, we compared the ribosome occupancy at each codon. The ribosome occupancy at a codon in the ribosomal A site (that is, the site accepting aminoacyl-tRNAs) is inversely proportional to the codon’s translational speed (59, 60). Therefore, in order to measure ribosome occupancy with single codon precision, we first calibrated the RPFs at the ribosomal A site, inferring the position of the A-site codon for each RPF, using the 3′end of the reads as established previously for bacterial RPFs (61). We considered 2,233 transcripts that passed our quality control criteria (*Materials and Methods*) and aligned them at their stop codons (UAA, UAG, UGA), resulting in a defined peak at the stop codon (position 0, Fig. 5 *B*, *Left*). We also observed a well-defined 3-nucleotide (nt) periodicity across the coding sequences followed by a loss of periodicity and a ribosome occupancy decline downstream of the stop codon (after position +25, Fig. 5*B*)—both features of genuine translation. We observed higher ribosome occupancy at proline codons (CCA, CCU, CCG, and CCC; Fig. 5 *C*, *Left*), corroborating commonly observed stalling at these codons (62, 63). As expected, these sites did not result in a drop of periodicity (Fig. 5 *C*, *Right*).

Overall, our analyses did not show a detectable variation of the ribosome occupancy at GidA-dependent codons (*SI Appendix*, Fig. S4 *A* and *B*). However, we rationalized that our analysis does not account for translation abortion events following the codons analyzed (64). To explore this, we assessed the ribosome occupancy in the flanking regions, i.e., downstream and upstream of the codons of interest (A site calibrated at ± 9, 12, 15, 18, and 21 nt distance, *SI Appendix*, Fig. S4 *C* and *D*). In the absence of U_34_ modification of the cognate tRNA (*tngidA* strain), the ribosome occupancy downstream of a single codon, the AGA codon, decreased markedly, indicative of ribosomal drop-off and aborted translation (Fig. 5*D*). We did not observe a tn*gidA* dependent drop off after UUA codons, but rather an increased ribosome occupancy in both the tn*gidA* and the tn*ladS* strains. Of note, AGA and UUA are both rare codons, occurring only 1,101 and 763 times, corresponding to a genome-wide frequency of 0.6 and 0.4 per 1,000 codons, respectively. Furthermore, we performed the same analysis on all codons (*SI Appendix*, Fig. S4 *C* and *D*). The leucine UUG codon has been defined as target of the GidA homologue (MnmG) in *E. coli*. However, its translation was not affected in our GFP reporter assay (Fig. 4*C*), nor did we observe any differences in ribosome occupancy up- or downstream of the UUG codon. Surprisingly, we detected an increased ribosome occupancy at isoleucine AUA codons in tn*gidA* (*SI Appendix*, Fig. S3 *C* and *D*). In *E. coli*, rare AGA codons have been reported to accumulate at the beginning of crucial genes (65). Likewise, AGA and, to a lesser extent, UUA codons clustered at the beginning of genes in *P. aeruginosa* PA14 (Fig. 5*E*). We also noted an accumulation of AGA codons preceding stop codons, with the highest prevalence at position −3 codons. These phenomena occur in core genes and do not apply to accessory genes. This observation may signify a form of codon usage fine-tuning to regulate protein production within the specific context of the translation machinery in *P. aeruginosa*. Furthermore, AGA codons appear either in close proximity to start or to stop codons, (Fig. 5*E*, VENN diagram). Of note, six genes with AGA codons located close to the start and/or stop codon, belong to the PseudoCAP function “Motility & Attachment” (*P* value ≤ 0.05). Together, our data suggest that impaired GidA-dependent modifications cause translation aberrancies preferably at AGA and not at UUA codons. The higher clustering of AGA codons and their higher usage than the UUA codon, might be the reason for the detectable global effects only at AGA codons.

### Impact of GidA on Protein Expression.

**Fig. 6.**
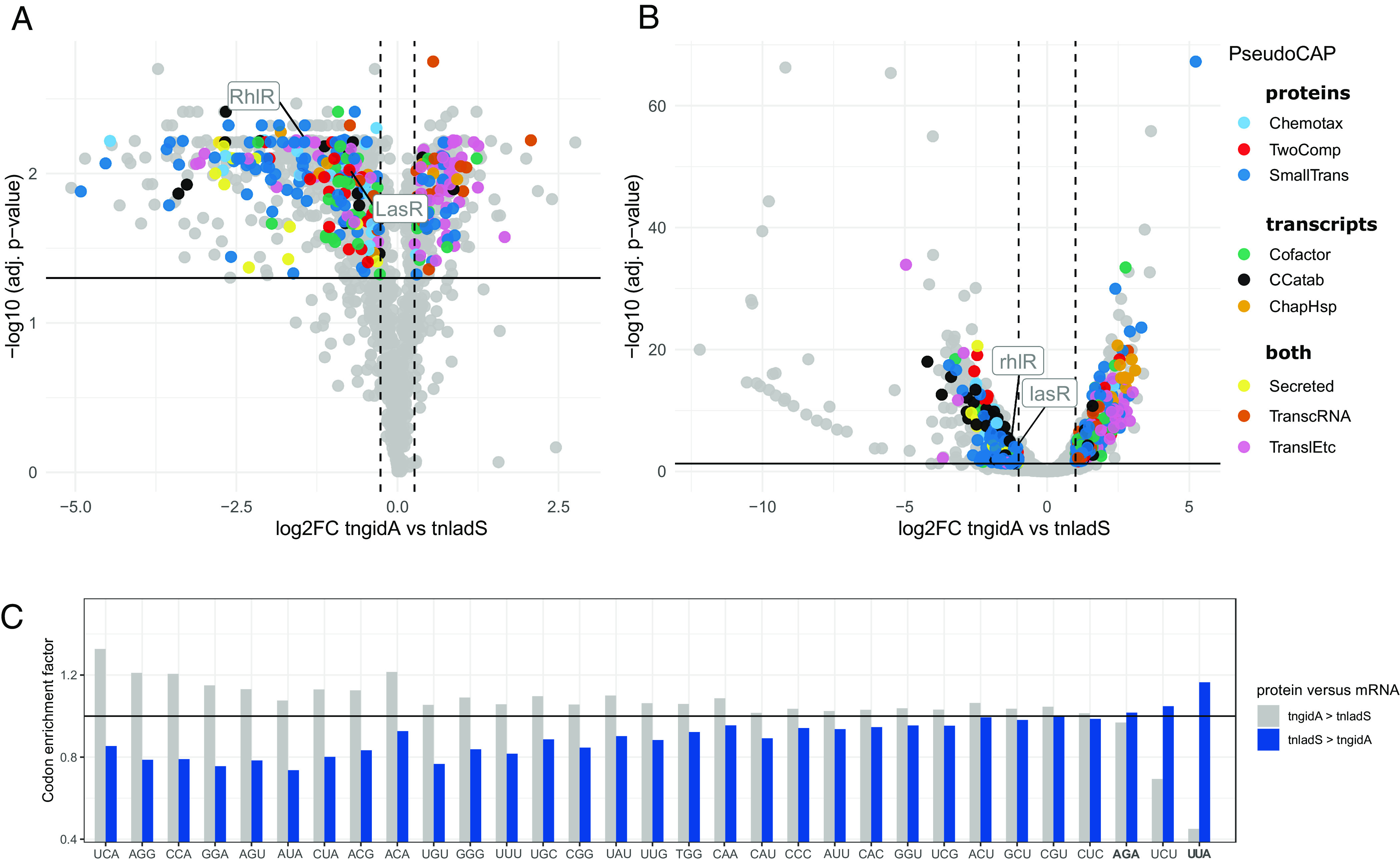
GidA-dependent global changes of protein and mRNA levels in *P. aeruginosa*. Volcano plots depicting the fold changes in protein (*A*) and mRNA (*B*) levels, comparing the PA14 tn*gidA* mutant and the tn*ladS* reference strain (n = 3). Proteins were considered differentially regulated at a ratio of tn*gidA*/tn*ladS* ≤ 0.83 or ≥1.2 (vertical lines) and Student’s *t* test *P* adjusted ≤ 0.05 (horizontal line). Transcripts were considered differentially regulated at a ratio of tn*gidA*/tn*ladS* ≤ 2 or ≥2 (vertical lines) and *P* adj. ≤ 0.05 (horizontal line). Functional PseudoCAP categories, significantly overrepresented in the group of differentially regulated proteins, are highlighted. Downregulated proteins were enriched in Chemotaxis, TwoComp (Two component regulatory systems), and SmallTrans (Transport of small molecules). Transcripts belonging to the enriched functions TranslEtc (translation, post-translational modification, degradation) and TranscRNA (transcription, RNA processing, and degradation) were upregulated in both, protein and transcript data, Secreted (secreted factors) genes were downregulated. The remaining enriched functions were only significant for the upregulated mRNAs (CCatab, ChapHsp, and cofactor). (*C*) Codon enrichment at gene groups level in proteome versus mRNA. Codon enrichment in protein groups with higher or lower protein versus mRNA levels, respectively in the tn*gidA* mutant compared to tn*ladS* strains. We generated lists of the top 25% genes with log twofold change of protein versus mRNA level higher in the tn*gidA* vs. tn*ladS* (tn*gidA* > tn*ladS*, in gray) and vice versa (tn*ladS* > tn*gidA*, in blue). The codon enrichment factor (*y* axis) is the ratio of genes containing a specific codon as compared to genes lacking that codon (enrichment factor). Significance was determined using hypergeometric testing. Codons with a codon enrichment score < 1.01 are not shown (*Materials and Methods*). AGA and UUA also had the strongest enrichment in an independent repetition experiment. Asterisks indicate Benjamini and Hochberg adjusted *P* values: *P ≤ 0.05, **P ≤ 0.01, ***P ≤ 0.001.

To determine the consequences of GidA-dependent tRNA modifications on protein composition, we employed the stable isotope labeling by amino acids in cell culture (SILAC) approach (66). This proteomic technique detected and quantified in total 2,334 proteins, of which 1,348 proteins were shared among all three replicates of the tn*ladS* and tn*gidA* mutants. Of these, 227 proteins were upregulated, and 628 proteins downregulated in tn*gidA* versus tn*ladS* (Fig. 6, FC ≤0.83 or ≥1.2 and FDR ≤ 0.05). Virulence and motility associated proteins were significantly less abundant in GidA-deficient cells. These included transport of small molecules, secreted factors (toxins, enzymes, and alginate), chemotaxis, and two component regulatory systems (Fig. 6*A* and Dataset S1). Additionally, the virulence and quorum sensing–related transcriptional regulators RhlR and LasR were downregulated in the tn*gidA* mutant. Highly abundant proteins in the tn*gidA* mutant strain were enriched for functional categories involved in basic cellular functions, such as transcription and translation (i.e. FDR ≤ 0.05). Of note, the latter two categories and the secreted factors were also enriched in the transcriptome (Fig. 6*B*). Chaperones and genes belonging to the biosynthesis of cofactors were up-regulated in the transcriptome only, whereas genes belonging to carbon compound catabolism were down-regulated.

To evaluate the role of specific codons, we considered the codon usage in the CDS of the proteins which were differentially expressed in the tn*gidA* and calculated an enrichment score (i.e. codon presence versus absence, Fig. 6*C*). We reasoned that codons that are abundant in proteins with a high protein-transcript-ratio (PTR, high translation efficiency: Enrichment score >1) would be less frequent in genes with a low PTR (low translation efficiency: enrichment score < 1), and vice versa. Indeed, the codons UUA, UCU, and AGA have an enrichment score > 1 for genes with a low PTR and an enrichment score of <1 for genes with a high PTR. However, this trend was not statistically significant.

In conclusion, our results support the notion that protein levels are modulated by the codon usage and the levels of modified tRNAs in the cell.

### Importance of *gidA* Gene Expression for the Virulence Phenotype of Clinical *P. aeruginosa* Isolates.

**Fig. 7.**
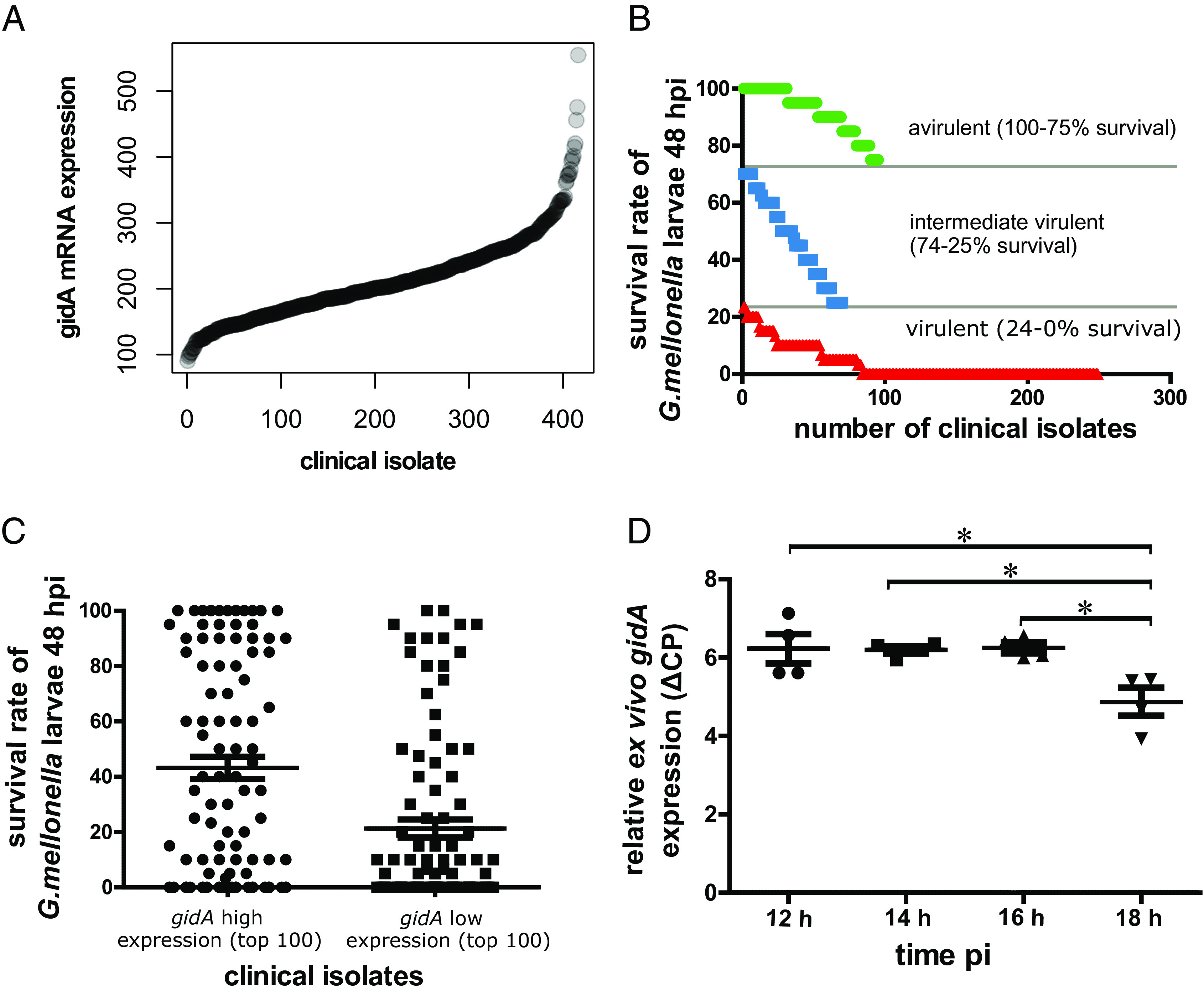
Importance of *gidA* expression on the virulence phenotype of clinical *P. aeruginosa* isolates. (*A*) Expression level of *gidA* in the transcriptome of 414 clinical isolates of *P. aeruginosa* isolates (OD 2 in LB) were sorted in ascending order. Gene expression was normalized (using trimmed mean of M-values and counts per million from the R package edgeR). (*B*) Survival rates of infected *G. mellonella* larvae 48 h pi (67). Clinical isolates marked in red correspond to virulent isolates (0 to 24% survival), isolates marked in blue correspond to intermediate virulent isolates (25 to 74% survival) and avirulent isolates are shown in green (75 to 100% survival). (*C*) The *G. mellonella* survival rates of those 100 clinical isolates, which expressed *gidA* at the highest and the lowest levels, respectively, are depicted. (*D*) Transcriptional activity of *gidA* ex vivo (after *G. mellonella* infection) measured by qRT-PCR relative to three housekeeping genes (PA14_07700, PA14_55650 and PA14_56080). Asterisks indicate *P* values as determined by the Mann–Whitney test: *P value ≤ 0.05.

We previously described that the virulence phenotype of 414 *P. aeruginosa* clinical isolates varies considerably in the *G. mellonella* (67). Given the strong impact of GidA on *P. aeruginosa* virulence in two in vivo models used in this study (Fig. 1), we evaluated whether variations in *gidA* expression levels (Fig. 7*A*) could explain differences in pathogenicity (Fig. 7*B*) (57). We built on previous work and took advantage of extensive transcriptome data that have been recorded for the 414 clinical isolates in early stationary phase (68, 69) and correlated *gidA* gene expression values with the virulence phenotype. Clearly, the clinical isolates that expressed low levels of *gidA* were generally more virulent (Fig. 7*C*), indicating that low *gidA* expression during early stationary growth phase is associated with higher pathogenicity. In light of our finding that the GidA protein is constitutively expressed throughout exponential growth and early stationary phase, but downregulated in late stationary growth (Fig. 2*H*), we analyzed *gidA* expression during the course of a *G. mellonella* infection. As depicted in Fig. 7*D*, *gidA* expression remained stable within the first 16 h of the larvae infection, but experienced a downshift after 18 h, suggesting that a dynamic expression, from a high expression in early infection to a downregulation at later time points, is important for the full pathogenicity phenotype.

In conclusion, our data indicate that not only the presence but also the dynamics in expression of *gidA* might be important for the establishment of the full *P. aeruginosa* pathogenicity. During early stages of infections, GidA seems to drive virulence factor production, while its downregulation at later stages facilitates translation of genes that are essential for bacterial survival and maintenance of infection.

## Discussion

The outrageous severity of acute infections, the increasing threat of multi-drug resistant isolates, and the recalcitrance of chronic biofilm-associated infections drives morbidity and determines mortality in the affected patients. In light of antibiotic refractory infections and non-sufficient effector mechanisms of the host immune system, the development of rational strategies to treat *P. aeruginosa* infections is urgently needed. Understanding the virulence mechanisms and their regulatory cascades is crucial in this context.

*P. aeruginosa* pathogenicity is multifactorial and affected by the conditional expression of a plethora of virulence genes. This presents a challenge to our efforts to pinpoint the most relevant virulence factors and, as a result, to search for innovative anti-virulence targets (pathoblockers). In this study, we highlight the essential role of *gidA* for *P. aeruginosa* pathogenicity in two in vivo model systems, thus corroborating previous observations on the impact of GidA on bacterial pathogenicity (34–38, 42). We found that the GidA-activated gene set is essential for the orchestration of the production of virulence determinants, cytotoxicity toward macrophages, biofilm recalcitrance, motility, and the adaptation to the stressful environment within the eukaryotic host. The *gidA* gene expression levels correlates with clinical isolates’ virulence in *G. mellonella*, underscoring the dominant contribution of *gidA* in determining pathogenicity and thus its importance as a promising pathoblocker target. However, since GidA is highly structurally homologous to the human mitochondrial enzyme MTO1, with relatively modest homology at the amino acid level (46.8%), further structural, functional, and medicinal chemistry studies are needed to ensure the specificity of putative novel antibacterial compounds.

Post-transcriptional chemical modifications are key modulators of tRNA decoding properties. Dependent on their specific modification and position in the tRNA molecule, they can have different effects. While modifications in the tRNA body mainly affect tRNA stability, tRNA modifications in the anticodon and anticodon loop influence both decoding efficiency and accuracy (20, 70, 71). Thus, synonymous codon choice greatly affects translation efficiency and influences protein levels hence the function of a gene (30, 72, 73). The codon bias and the impact of U_34_ tRNA modifications have been extensively studied in eukaryotic cells (20, 31, 33, 60, 72, 74, 75). Mounting evidence places hypomodified tRNAs and changes in tRNA abundance more centrally in establishment and progression of human pathologies, such as cancer, diabetes, and neurological disorders (30–33, 74, 76–78). In contrast, there have been limited attempts to unravel the link between tRNA modifications and bacterial phenotypes via their tuning effects on gene expression (79, 80).

Our approach, which combines mass spectrometry to identify tRNA modification and the use of translational reporter fusions, with cell-wide approaches to analyze codon usage and gene expression (i.e., measurement of translation with codon precision by ribosome profiling and quantification of mRNA and protein levels by RNA-Seq and proteomics, respectively), led to the finding that GidA alters the translation efficiency at selected *P. aeruginosa* codons. As a result, proteins that are enriched with *gidA*-dependent codons, such as AGA and UUA, are significantly less abundant in the proteome of the *gidA*-deficient cells. Among them were many virulence- and motility-associated proteins.

We did not observe any increase of ribosome occupancy at AGA or UUA positions in the absence of GidA. The low codon usage of these codons may mask the effect on translatome-wide scale. However, we observed a ribosomal drop-off at AGA codons. We further show that AGA codons are located mainly in the downstream vicinity of start and upstream of stop codons. AGA codons in the start codon vicinity could trigger an early abortion of translation and a resource-saving mechanism. It has been described that translation termination fidelity requires a ribosomal slow-down upstream of the stop and that sequence context in the close proximity of the native stop codons defines the fidelity of termination (81). Specifically, codons encoding for bulky positively charged amino acids, such as arginine, are preferred at positions −2 upstream of the stop codon (82). An improper decoding of AGA at such impactful position may impair translation termination and consequently lead to C-terminally extended aberrant proteins.

We observed a modest decrease in ribosome occupancy of UUA containing mRNA, but no *gidA*-dependent changes in ribosome occupancy at the codon level. We also did not observe a drop off after UUA codons. This could be explained by the much lower abundance of UUA codons and consequently compared to AGA their lower enrichment in regions crucial for translation. Nevertheless, we observed a lower abundance of proteins enriched in UUA codons. Lack of posttranslational tRNA modifications fails to maintain a proper reading frame and triggers frameshifting (83). Since we cannot detect this in the RiboSeq data, we cannot exclude the possibility that frameshifting contributes to the production of aberrant proteins.

Overall, our results suggest that although both codons, AGA and UUA, rely on GidA for translation, the transcript outcome varies considerably depending on the codon identity and its position in the genome. In the same line, the magnitude of ribosome stalling triggered by hypomodified tRNAs differs between organisms. For example, loss of mcm^5^U_34_ or ncm^5^U_34_ modification had minor effects in yeast both during normal growth and when exposed to stress but exhibited much larger effects in nematodes (32). The effects of subtle ribosome stalling may be amplified by additional species-specific factors, such as tRNA abundance, mRNA structure, or genomic background. This obviously includes the uneven distribution of AGA and UUA codons, which was also previously described for *E. coli* (65). In this context, it is interesting to note that the unequal codon distribution at the beginning and at the end of the genes is mainly found in the *P. aeruginosa* core genes and to a much lesser extent in the horizontally acquired genes of the *P. aeruginosa* accessory genome. This implicates a fine-tuned post-transcriptional control of gene expression based on the species-specific use of rare codons, whose decoding depends on the activity of *gidA*, which itself is subject to regulation.

We show that the GidA enzyme is stably expressed across a number of different environmental conditions, while we observed a down-shift in its expression levels during stationary growth. We also found a stable high *gidA* gene expression during early phases of a *G. mellonella* infection. While this may drive the establishment of the infection via a positive impact on virulence factor production, the downregulation of *gidA* at later stages might facilitate translation of genes that are essential for bacterial survival and thus maintenance of the infection. In this context, it is interesting to note that we found a significant correlation of low *gidA* levels in in vitro stationary phase cultures of clinical isolates with a high *G. mellonella* killing activity. This indicates that especially the down-shift of *gidA* expression levels at later stages plays a key role in determining the pathogenicity potential. This appears to vary among clinical isolates, raising the possibility of evolving the pathogenicity capacities by dynamically changing GidA levels.

Taken together, our study outlines emerging mechanisms of post-transcriptional regulation that involves modulation of tRNA modifications to alter expression of drivers of bacterial pathogenicity in a codon-biased fashion. We found that tRNA modifications that impact decoding of specific codons directly modulate the expression efficiency of target transcripts which are enriched for those codons. Of note, we have focused on GidA-mediated *P. aeruginosa* phenotypes. However, our approach of profiling the consequences of chemical tRNA modifications is wide-reaching for others yet uncharacterized tRNA modifications in *P. aeruginosa* and in other bacterial species. Studies on how we can interfere with bacterial adaptation to the host niche will continue to be of importance, as misuse of valuable antibiotics has resulted in increased levels of resistance, a major global health issue. Targeting the GidA-orchestrated molecular pathways that drive disease outcome might provide a unique opportunity to spur clinical pipelines for development of innovative treatment options and alternative antimicrobial therapies.

## Materials and Methods

Detailed information on the materials and methods used for bacterial culture conditions, motility, *G. mellonella* virulence, biofilm formation, antibiotic susceptibility testing, and cytotoxicity assays can be found in *SI Appendix*. Protocols for our transcriptional, proteomic, nano-tRNA-seq, and ribosome profiling experiments are also provided as well as the methods describing LC–MS analysis for the quantification of total or purified individual tRNAs modifications. Methods for the quantification of GidA protein (by multiple reaction monitoring) and *gidA* (by ex vivo RT-PCR) can be found as well as the description of assays to measure translation efficiency.

## Supplementary Material

Appendix 01 (PDF)

Dataset S01 (XLSX)

## Data Availability

RNA-seq, Nano-tRNAseq, Scripts used for base calling, alignment, and subsequent processing steps data have been deposited in NCBI GEO database (GSE149306) (57), European Nucleotide Archive (PRJEB69610) (55); GitHub (https://github.com/DepledgeLab/tRNA-studies) (56), respectively.
