# Supplementary material for: tRNA epitranscriptome determines pathogenicity of the opportunistic pathogen Pseudomonas aeruginosa
Source: Proc Natl Acad Sci U S A. 2024 Mar 7;121(11):e2312874121. doi: 10.1073/pnas.2312874121 (PMC10945773; doi:10.1073/pnas.2312874121)
Supplement: Supplementary file 1 — Appendix 01 (PDF) [file pnas.2312874121.sapp.pdf]

**Supplemental information (SI)**

## Material and methods

### *Bacterial strains, media, and culture conditions*

Strains, plasmids, and primers used in this study are listed in Table S2 and Table S3. All chemicals were purchased from Sigma-Aldrich or Carl Roth unless otherwise stated. Strains were cultivated in lysogeny broth (LB) medium or in modified M9 medium (42.2 mM $Na_2HPO_4$, 22 mM $KH_2PO_4$, 18.7 mM $NH_4Cl$, 8.6 mM NaCl, 1 mM $MgSO_4$, 0.1 mM $CaCl_2$, 20 mM Glucose, 10 µM Fe(II)sulfate, and 0.1 mM of each of the 20 canonical amino acids except lysine (1.368 mM)). Carbenicillin was used at a final concentration of 400 $\mu g.ml^{-1}$, gentamycin at a final concentration of 50 $\mu g.ml^{-1}$, and tetracycline at a final concentration of 100 $\mu g.ml^{-1}$. The *P. aeruginosa* clinical isolates analyzed in this study were collected at five major sampling sites from clinical microbiology laboratories of hospitals across Germany and at sampling sites in Spain, Hungary and Romania. In addition, *P. aeruginosa* isolates were collected in German private practice laboratories or provided by strain collection curators. For all of these isolates the genomic DNA and RNA have been sequenced (bactome.helmholtz-hzi.de, [1]).

### *Cytotoxicity*

RAW264.7 (ATCC number: TIB-71TM) cells were cultured in DMEM (4.5 $g.l^{-1}$ glucose) and supplemented with 10% FCS, 20 mM HEPES and 1% BSA at 37°C, 5% $CO_2$. Eukaryotic cells were seeded in DMEM at a concentration of 5 x $10^5$ cells in a 24-well plate. Bacterial overnight cultures were sub-cultured at a 1:30 dilution and grown for 3.5 h to mid-log phase. Following growth, bacteria were washed with PBS and adjusted to MOI 1 (5 x $10^5$bacteria per 100 µl) in phenol-red free DMEM. RAW macrophages were infected with 5 x $10^5$ (MOI 1) bacteria of the respective strain in phenol-red free DMEM media. The supernatant was collected every hour for 4 h. LDH levels were measured using the CytoTox 96 Non-radioactive Cytotoxicity Assay (Promega) according to the manufacturer protocol. PBS served as negative, viability control, and 10% (v/v) Triton-X100 as killing control.

### *Targeted LC-MS analysis to quantify tRNA modified uridine derivatives*

Small RNA isolation and LC-MS/MS analysis were performed as previously described [2]. Briefly, small RNA fraction was purified and 30 µg RNA were enzymatically hydrolyzed into nucleosides (adapted from [3]). Tenofovir was added as Internal Standard (IS, final 50 $ng.ml^{-1}$). Nucleosides were analyzed by reverse-phase chromatography coupled to a Linear Ion Trap Quadrupole (QTRAP5500, Sciex). Quantification was done by external standard calibration with synthetic standards of modified ribonucleosides as reference. The Analyst® 1.6 Software (Sciex) was used for data analysis. The extent of modification in total tRNA was expressed as the ratio of target modification to total canonical ribonucleosides (mol/mol) and is represented as modifications per 1000 ribonucleosides. Data were visualized with GraphPad Prism® 6 (GraphPad Software, Inc., La Jolla, CA, USA). Statistical significance was determined using student´s t-test with correction for multiple comparisons (Holm Šídák). The experiment was performed with three biological and two technical replicates.

### *GFP-based translation efficiency assay*

Artificial codon repetition sequences for arginine (AGAAGAAGAAGA, AGGAGGAGGAGG), glycine (GGAGGAGGAGGA, GGGGGGGGGGGG), leucine (TTATTATTATTA, TTGTTGTTGTTG) and mixed codons (AGAGGAAGAGGA, AGAGGATTAAGGGGGTTG, CGTGGTCTCCGTGGTCTC) were designed, fused to a superfolded GFP (*sfGFP*) encoding DNA fragment and cloned into the pME6032 vector. The respective constructs as well as the empty control vector were transformed into the reference strain PA14 tn*ladS* and PA14 tn*gidA*. For the detection of the GFP signal, 200 µl late exponential growth phase culture was

transferred into a well of a black 96 well plate. The GFP was excited at wavelength 485 nm and GFP-emission wavelength of 528 nm was detected using an FLx800 fluorescence reader (BioTek). Samples were measured in four technical and three biological replicates. The GFP signal was normalized to the $OD_{600}$ and the non-IPTG induced signal was subtracted from the IPTG induced signal.

***Purification of individual tRNA from total tRNA***

Individual tRNAs were purified using a selective hybridization approach as described previously [2, 4]. Magnetic Beads (Dynabeads-M280 Streptavidin, Thermo Fisher Scientific) were prepared according to the manufacturer's instructions. First, the beads were suspended in 1 ml 2x B&W (binding & washing buffer) consisting of 10 mM Tris-HCl pH 7.5, 1 mM EDTA, 2 M NaCl. After washing of the beads, a highly specific DNA oligonucleotide complementary to the target tRNA (Table S3) was attached according to the manufacturer's instructions. Total tRNAs (100 µg) were mixed with an equal volume of 2x hybridization buffer (20 mM Tris-HCl pH 7.5, 1.8 M TMACl, 0.2 mM EDTA) and incubated at 65°C for 10 min with gentle mixing. The beads were washed three times (10 mM Tris-HCL pH 7.5) and mixed with total tRNAs, followed by 10 min incubation at 65°C and 3h at room temperature with gentle mixing. Afterwards, the unbound tRNA was removed and the hybridized resin washed three times. The target tRNA was eluted by suspension in 30 µl Tris-HCl (10 mM, pH 7.5) and incubation at 75°C for 5 min to detach the biotin-streptavidin-binding. The supernatant was transferred to a new tube an immediately placed on ice, followed by addition of 1/10 volume of sodium acetate and 2.5× volumes of ethanol and incubated at -20°C overnight. On the next day, the tRNA was recovered by centrifugation at 15 000 x g for 30 min at 4 °C. The tRNA pellet was washed twice with ethanol (70 % v/v), dried and resuspended in nuclease-free water. The tRNA concentration was determined in a plate reader (Agilent, Take3 Micro-Volume plate) and RNA quality was checked using a Bioanalyzer (Small RNA Kit, Agilent, Waldbronn, Germany).

***Virulence factor assays***

Pyocyanin production was quantified as described previously [5]. Briefly, bacterial main cultures were grown for 24 h in LB. Supernatant (5 ml) was extracted with 5 ml chloroform. Then, 3 ml of the organic phase were mixed with 1 ml 0.2 M HCl, vortexed and the absorbance of the aqueous phase was determined at 520 nm. For quantification of pyocyanin ($µg.ml^{-1}$) the absorbance value was multiplied by factor 17.072. Values were normalized by $OD_{600}$ of the culture. Rhamnolipid production was monitored using the orcinol based assay as described previously [6]. Overnight LB cultures were used to inoculate 24 h main cultures in normal LB. 600 µl diethyl ether were added to 300 µl supernatant to extract rhamnolipids. Extraction was repeated two more times and the combined organic phases were dried. The pellet was resuspended in 100 µl water, 100 µl 1.6 % orcinol, and 800 µl 60 % $H_2SO_4$. The mixed solution was then incubated for 30 min at 80°C. Absorbance was measured at 421 nm and a calibration curve was used to calculate rhamnolipid concentrations. Rhamnolipid concentrations were normalized to the $OD_{600}$ of the culture.

***Motility assays***

Swimming, swarming, and twitching assays were performed as described previously [73]. Swimming and twitching behavior was monitored and recorded after 48 h, swarming after 24 h.

***Microscopic biofilm images***

Biofilm characteristics were monitored by the use of confocal microscopy as described previously [7]. Overnight LB cultures were adjusted to an $OD_{600}$ of 0.05 and 100 µl of this suspension were transferred

into a well of a microtiter plate (96 well, half-area, Greiner). For each strain, five biological replicates were prepared. The microtiter plate was covered with an air permeable foil and incubated at 37°C for 24 h in humid atmosphere. LIVE/DEAD staining (BacLight Bacterial Viability kit, Molecular Probes/Invitrogen) was applied, and the strains were incubated for an additional 24 h before images including z-stacks were acquired using a confocal microscope (TCS SP8, Leica Microsystems) with a 40x/NA 1.1 water-immersion objective. 3D images were visualized with IMARIS software package (version 5.7.2, Bitplane).

***Antimicrobial susceptibility testing of planktonic and biofilm-grown cells***

*P. aeruginosa* biofilms were grown in LB medium for 24 h in 96-well plates as described [8] and treated with ciprofloxacin for an additional 24 h, before the surviving bacteria were determined by counting the colony forming units (CFU). MIC values of planktonic cultures were determined by broth microdilution and visual inspection of growth inhibition after 18 h.

***Galleria mellonella virulence assay***

The *Galleria mellonella* model system was used to determine *P. aeruginosa* virulence as described previously [9]. Briefly, bacterial strains were grown in LB medium to an $OD_{600}$ of 2-3. Cells were pelleted by centrifugation at 8000 x g for 3 min and resuspended in PBS (10 mM, pH 7.4). Then, 20 µl containing 100 CFU were injected into the haemocoel of the hindmost proleg of the larvae. The infected larvae were incubated in the dark at 37°C. Mortality rates of 10 replicate larvae were monitored for 72 h in at least three independent experiments. Mortality was asserted by visual observation of melanization of the cuticle and lack of movement after stimulation.

***In vivo competition assay***

A 1:1 mixture of PA14 tn*ladS* and tn*gidA* strains dilutions (2.5 x $10^6$ CFU per strain) was injected intravenously into mice containing a subcutaneous CT26 tumor [10]. At the same time, 100 µl of this mix was used to inoculate 10 ml LB culture. The infection dose was controlled by plating dilutions of the mix followed by CFU counting. After 48 h, the mice were sacrificed and the tumor tissue was dissected and homogenized. Different dilutions of the homogenate and of the 48 h LB culture were plated on agar plates and incubated overnight at 37 °C. The PA14 tn*ladS*/tn*gidA* ratio of *in vivo* and *in vitro* experiments was determined by testing 28 randomly selected colonies per replicate by colony PCR. The ratio was calculated as follows: $\sum_{i=1}^{n} \# PA14\ tnladS / \sum_{i=1}^{n} \# PA14\ tngidA$ with *n*(*in vitro)*=5, *n*(*in vivo)*=9.

***Transcriptome analysis***

*P. aeruginosa* cultures (in duplicates) were grown to $OD_{600}$ of 2 and RNA was extracted from cells with the RNeasy Mini Kit (Qiagen) in combination with Qiashredder columns (Qiagen) according to the manufacturer's instruction. Ribosomal RNA was removed using the Ribo-Zero Bacteria Kit (Illumina) and the ScriptSeq v2 Kit (Illumina) was applied to generate cDNA libraries. Single end mode was used for sequencing on an Illumina HiSeq 2500 device involving 50 cycles. The *stampy* [76] software and the R package DESeq2 [77] was applied to map the sequences to the PA14 reference genome and to analyze differential gene expression. To define differentially expressed genes adjusted *p*-values were calculated as described by Anders and Huber [78]. The raw sequencing files have been deposited in the NCBI GEO database under the accession number GSE149306.

**Nano-tRNAseq library preparation**

Total tRNA pools purified from tn*ladS* and tn*gidA* strain were deacylated prior to preparation for Nano-tRNAseq. tRNAs were resuspended in 20 µl of nuclease-free water, mixed with 190 µl of 100 mM Tris-HCl (pH 9.0), and incubated at 37°C for 30 min. Deacylated tRNAs were purified using Zymo RNA Clean and Concentrator-5 kit (Zymo Research, R1017) according to manufacturer´s instructions. tRNA integrity was confirmed using Agilent Bioanalyzer (Agilent, Waldbronn, Germany).

tRNA libraries were generated following the Nano-tRNAseq protocol published by Lucas *et al.* (2023) using the SQK-RNA002 kit from Oxford Nanopore Technologies (ONT) with modifications to the protocol outlined below [11]. Oligonucleotides used for nano-tRNAseq were obtained from Integrated DNA Technologies (IDT). The 5′ RNA splint adapter (/5/rCrCrUrArArGrArGrCrArArGrArArGrArArGrCrCrUrGrGrN, with N depending on the respective complementary canonical base for tRNAs) was specifically designed to complement the 3′ NCCA overhang of mature tRNAs, while the 3′ splint RNA:DNA adapter (/5Phos/rGrGrCrUrUrCrUrUrCrUrUrGrCrUrCrUrUrArGrGrArArArArArArArArAAAA) was designed to hybridize with the remaining portion of the 5′ RNA splint adapter, featuring a short poly(A) segment to facilitate annealing with the RTA adapter. The 5′ and 3′ splint adapters were mixed at an equimolar 1:1 ratio in 10 mM Tris-HCl buffer (pH 7.5), containing 50 mM NaCl, and 1 µl of RNasin Ribonuclease Inhibitor (Promega, N251A), achieving a final concentration of 50 ng.µl$^{-1}$. The mixture was heated to 75°C for 15 s and gradually cooled down to 25°C at a rate of 0.1°C.s$^{-1}$ to promote adapter hybridization. DNA oligonucleotides with identical sequences to the ONT RTA adapters were procured from IDT and annealed in the same manner as the 5′ and 3′ splint adapters.

Next, deacylated total tRNAs were ligated to the pre-annealed 5′ and 3′ splint adapters at a molar ratio of 1.2:1. The ligation was carried out at room temperature for 2 h in a total reaction volume of 50 µl. The reaction mixture contained 20% PEG 8000 (NEB, B10048), 1× T4 RNA Ligase 2 Buffer (NEB, B0239S), 2 µl T4 RNA ligase 2 (10.000 U.mL$^{-1}$, NEB, M0239L), and 1 µl of RNasin Ribonuclease Inhibitor (Promega, N251A). Subsequently, a 2× volume of room-temperature-equilibrated AMPure RNAClean XP beads (Beckman Coulter, A63987) was gently pipetted into the reaction and incubated for 15 min at room temperature with occasional light tapping. Afterwards, the beads were washed with freshly prepared 70% ethanol and left to air dry. The samples were eluted by resuspending the beads in nuclease-free water and incubated for 10 min at room temperature. The RNA concentration was determined using RNA HS Qubit Fluorometric Quantification (Thermo, Qubit RNA BR Assay Kit, Q10210).

Afterwards, 200 ng of 5′ and 3′ ligated tRNAs were ligated to the pre-annealed RTA adapters at a molar ratio of 1:2. The ligation was carried out at room temperature for 30 min in a total reaction volume of 15 µl, consisting of 1× Quick Ligation Reaction buffer (NEB, B6058S), 1.5 µl of T4 DNA Ligase (NEB, M0202M, 2,000,000 U.ml$^{-1}$), and 0.5 µl of RNasin Ribonuclease Inhibitor (Promega, N251A). Following ligation, a reverse transcription master mix composed of 13 µl of nuclease-free water, 2 µl of 10 mM dNTPs (NEB, N0447S), 8 µl of 5× Maxima H Minus Reverse Transcriptase Buffer, and 2 µl of Maxima H Minus Reverse Transcriptase (Life Technologies, EP0751) was added directly to the ligation reaction and incubated at 60°C for 1 h, followed by heating up at 85°C for 5 min, and cooling down to 4°C on ice. The linearized tRNAs were purified using 2× AMPure RNAClean XP beads, following the same procedure as the ligation reaction.

Finally, ONT RMX sequencing adapters were ligated to the purified tRNAs at room temperature for 30 min in a total reaction volume of 40 µl. The reaction mixture included 1× Quick Ligation Reaction

buffer (NEB, B6058S), 3 µl of T4 DNA Ligase (NEB, M0202M, 2,000,000 $U.ml^{-1}$), and 6 µl of RMX adapters. Subsequently, a 2× volume of AMPure RNAClean XP beads was added to the reaction by gently pipetting up and down, and incubated for 10 min at room temperature with occasional light tapping. Samples were washed twice with 150 µl of WSB (Wash Buffer). Elution of the recovered tRNAs was performed in 20 µl of ELB (Elution Buffer), and the sample was incubated for 10 min at room temperature. The final library was prepared by adding 17.5 µl of nuclease-free water and 37.5 µl of vortexed RRB, and kept on ice until loading. The MinION flow cell (FLO-MIN-106) was quality controlled, primed, and loaded following the standard ONT SQK-RNA002 protocol.

**Nano-tRNA-Seq data processing and analysis**

The sequencing run was performed for 24 h without live basecalling and bulk output files were obtained for each run. Fast5 data were basecalled in high-accuracy mode without trimming using Guppy v6.5.7 (https://community.nanoporetech.com/downloads) and aligned against a tRNA database with BWA mem v0.7.17 (https://doi.org/10.48550/arXiv.1303.3997) using the Nano-tRNASeq parameters recommended [11]. SAMtools v1.15 [12] was used in downstream processing to retain only primary alignments with a mapQ score greater than 0 in the forward orientation [ samtools view -F 2324 -q 1 ] and to sort and index datafiles. Abundance counts for each tRNA were generated using a simple one-liner [ samtools view infile.bam | cut -f3 | grep -v LN | sort | uniq -c | sed "s/^[ \t]*//" | sed "s/ / \t/g" ] and scatter plots generated using the ggplot2 package [13] in R studio with R v4.1.1. Differential expression analysis was performed similarly using the DESeq2 package [14].

The nanopore sequencing datasets associated with this study are available as basecalled fast5 files via the European Nucleotide Archive under the accession PRJEB69610. The scripts used for base calling, alignment, and subsequent processing steps are available at https://github.com/DepledgeLab/tRNA-studies.

***Ribosome profiling***

Ribosome profiling was performed in triplicate for each strain, as described previously, with some modifications [15, 16]. Briefly, single colonies of tn*ladS* or tn*gidA* CFU were grown over night in 10 ml of LB supplemented with 50 $µg.ml^{-1}$ gentamycin. The preculture was used to inoculate 200 ml of pre-heated LB and incubated at 37°C, 180 RPM, until $OD_{600}$ reached 0.4 and harvested by rapid filtration through a nitrocellulose filter 0.2 µm (Carl Roth HP40.1), scraped off the filter, transferred into a 1.5 ml tube and flash-frozen in liquid nitrogen. Next, 5 µl lysis buffer per mg of pellet were used to resuspend the pellet [20 mM HEPES pH 7.6 (Millipore L1613), 6 mM magnesium acetate (Sigma 63052), 30 mM potassium acetate (ThermoFisher J63372), 4 mM β-mercaptoethanol (Sigma M6250), 100 U/ml DNase I (Roche), 100 U/ml Ready-lyse lysozyme (Lucigen R1804M)] and incubated 5 min on ice. Lysates were clarified by centrifugation, supplemented with 15 mM $CaCl_2$, 0.5 $U.µl^{-1}$ SuperaseIn (Invitrogen AM2696), chloramphenicol 0.1 $mg.ml^{-1}$ (Sigma R4408). An equivalent of 8 units $A_{\lambda=260}$ was digested with 1000 Units of MNase (NEB M0247) at 25°C, 1000 RPM, for 30 min. A control sample was treated identically without MNase to serve as a control for polysome profiling. Digestion by MNase was stopped by addition of EGTA at 6 mM final concentration. Lysates were layered onto 10-50% linear sucrose gradient prepared with a Gradient Master (BioComp) in polysome buffer [20 mM HEPES pH 7, 6 mM magnesium acetate, 30 mM potassium acetate, 4 mM 2-mercaptoethanol]. Following centrifugation for 3 h at 41.000 RPM, 4°C in a SW 41-Ti rotor, sucrose gradient were fractionated (Piston Gradient Master, Biocomp), fractions containing monosomes were collected, and immediately

frozen in liquid nitrogen. RNA footprints were isolated from monosome fractions with Tri-Reagent (Zymo clean and concentrator). Total bacterial RNA from the same culture was extracted using the RNeasy Mini Kit (74106, Qiagen). DNA was removed with a DNA-free DNA removal kit (Invitrogen) and depleted for rRNA and ribosomal RNA using our previously published method [17]. RNA fragments between 15 – 45 nt were extracted from denaturing 15% polyacrylamide gels using for reference a small RNA Marker (Abnova R0008). Size-selected RNA was converted to cDNA libraries using a NEBNext Multiplex small RNA library prep for Illumina (E7300) using custom multiplex primers. Sequencing was performed on an Illumina HiSeq 2500 platform as a 50 bp paired-end sequencing with 30 million reads per sample at the Genome Analytics Research Group in the Helmholtz Centre for Infection Research (HZI) of Braunschweig.

The sequencing depth of three replicate samples of the ribosomal footprint of tn*gidA* and tn*ladS* was between 37.3 and 41.5 million reads. Adapters were removed using cutadapt version 2.8 [18]. Trimmed reads with a length shorter than 22 nt were discarded. Mapping to the *P. aeruginosa* PA14 (NC_008463.1) reference genome was performed using bowtie2 version 2.3.5.1. [19] using the option "--very-sensitive-local". Differences in ribosome occupancy between the tn*gidA* mutant and the reference isolate were calculated on the gene level using the R package DESeq2 [14] comparing (tn*gidA*_RBF - tn*gidA*_TR) - (tn*ladS*_RBF - tn*ladS*_TR). Genes with less than 1 count per million (cpm) in at least 2 samples were excluded from the analysis. For codon enrichment analysis, genes were ordered by their adjusted p value and the top 25% genes with the highest and the lowest ribosome occupancy (log2 fold change higher/lower 0) in tn*gidA* were selected. Enrichment analysis was performed for each codon using the R function phyper and information about presence/absence of codons within each gene. The codon enrichment score (fold enrichment of the number of genes bearing a codon compared to the number of genes expected by chance) was calculated using the formula Cs/Cb, whereas Cs is the proportion of these gene-subsets containing a codon and Cb is the codon proportion in the total number of genes used in the DESeq analysis.

For data analysis at codon level, the ribosome occupancy was assigned to the 3' end of the RPFs. Therefore, RPFs were grouped by lengths and each 3' nucleotide of the groups was aligned to the stop codons to determine the offset for the center nucleotide in the A-site, the 3'-end anchored reads were plotted for each read length and the offset in nucleotides was defined as the distance between the stop codon and the highest peak downstream of the stop codon [20]. For further analyses, the reads with a length of 40 and 41 nt were used with an offset of 16 and 17 nt, respectively. For P site assignment, the offset was 19 and 21 nt, respectively.

3' assigned RPFs were written into Artemis files that contain counts per genome position and strand. These files were normalized using counts per million (cpm) and the weighted trimmed mean of M-values with singleton pairing (TMMwsp, edgeR functions calcNormFactors and cpm). Protein-coding genes with a gene length of > 150 nt were considered for the following analysis. To control for varying mRNA levels, genes (including 50 nt upstream/downstream of start/stop codons) were individually normalized (R function scale without centering). The codons and the 50 preceding or following nucleotides were arranged one below the other and mean values for each of these nucleotides were calculated and plotted to visualize the coverage of the 3' end of each read.

To compare the ribosome occupancy of different codons at the A site, the normalized reads for the codons and their 50 nt downstream and upstream nucleotides (see above) were used as the basis to calculate the differences at each codon site. Then, codons with no reads in all samples were removed

from the dataset and the mean value for each codon position and the mean value for each sample were calculated. To examine the distribution of each codon at the different positions, we calculated the mean reads for each codon position and the mean of the three replicates. To estimate a possible ribosome drop off, we compared the downstream and the upstream regions with respectively 9,12,15,18, and 21 nt distance to the respective codon (P site). Similar to the codons itself, we calculated the mean from the different positions or replicates, respectively, and then calculated the difference between tn*gidA* and tn*ladS* mutants. Data were plotted using the R library ggplot2 [13].

***LC-MS- based proteome analyses***

For isotopic labeling-based proteome analysis, PA14 tn*ladS* and PA14 tn*gidA* were grown to an $OD_{600}$ of 2. As previously described, to generate heavy labeled proteins as an internal standard, non-lysine metabolizing PA14 tn*ldcC* cells were grown in M9 medium containing stable isotope labeled lysine ($^{13}C_6{}^{15}N_2$-L-lysine (lys8), 1.368 mM, SILANTES) to an $OD_{600}$ of 2 [52]. Before protein extraction, the tn*ladS* and the tn*gidA* cells were mixed 1:1 with lys8 labeled tn*ldcC* "spike-in" standard. Cells were lysed by sonication in SDS-lysis buffer (20 mM TRIS-HCl, pH 6.8, 2% SDS, 6.8% glycerol, 20 mM DTT, 1x Protease inhibitor (Roche). SDS-PAGE was used for pre-fractionation and in-gel LysC (Wako Chemicals) digestion was applied. Peptides were subsequently measured by the combined use of a reversed phase nanoflow ultrahigh pressure liquid chromatography (RSLC) system (Thermo Fisher Scientific) and an LTQ Orbitrap Velos mass spectrometer (Thermo Fisher Scientific). To Identify and quantify proteins, raw files generated by LC-MS/MS analysis were processed using the MaxQuant software package (version 1.5.2.8) [21] with the implemented search engine Andromeda [22]. A custom-made database of *P. aeruginosa* strain PA14 annotated entries of UniProt database and the Pseudomonas Genome Database [23, 24] was used. Only proteins that were simultaneously identified in three replicates were used for further analysis. Student's t-test was used to assess significance in protein abundance between different strains.

***GidA quantification by LC-MS-based Multiple reaction monitoring (MRM)***

Cellular GidA levels were determined in the PA14 tn*ladS* strain following growth under various environmental conditions. These included growth in M9 medium, in cultures subjected to four hydrogen peroxide (1 mM) treatments in 30 min intervals starting at 7 h post inoculation, and attached to the bottom of petri dishes. The cultures in petri dishes were incubated at 37°C for 2 h, non-attached cells were removed by several washing steps with ice-cold PBS. Control cells were incubated for 2 h at 37°C and 180 RPM. Harvested cells were mixed with the $^{13}C_6{}^{15}N_2$-L-lysine labeled PA14 tn*ldcC* strain grown for 7.5 h at 37°C and 180 RPM. For the targeted LC-MS analysis mixed samples were handled as described above. However, only the gel area between 60-80 kDa was used for digestion by Trypsin (Promega). Peptides were separated applying a 50 min HPLC (Ultimate 3000, RSLCnano system, Thermo Scientific) 2-80 % acetonitrile gradient using a flow rate of 250 nl and a 2 cm trap column (3 µm C18 particle, 2 cm length, 75 µm ID, Acclaim PepMap, Thermo Scientific) in combination with a 25 cm long reversed phase separation column (2 µm C18 particle, 75 µm ID, Acclaim PepMap, Thermo Scientific). Peptides were transferred into the 4000 QTrap triple quadrupole mass spectrometer (Sciex) by electrospray ionization using a HPLC column connected emitter (NewObjective). Settings for QTrap were as follows: The curtain gas was set to 12, collision gas to high, ionspray-voltage to 2800 V, ion source gas 1 to 4, ion source gas 2 to off, interface heater temperature to 150°C, declustering potential to 80, entrance potential to 10, and collision cell exit potential to 15. Collision energy was optimized for every transition. MRM methods for two peptides of each of the target proteins (GidA and four housekeeping proteins) were created with MRMPilot 2.1 (Sciex) (PA14_73370 (GidA): WAAFEAK,

IPGVTPAAISLLLIHLK; PA14_54450 (NadB): FSVLASGGASK, LLSEIDEFYSNY; PA14_07130 (TktA): AAAYVADVANK, IAIEAAHADYWYK; PA14_62970 (DnaK): MYAEQAQQGED, IELSSTQQTDV; PA14_00090 (GlyS): ALNSLGEAFLSGIEK, FITVANVESK). For each peptide two to three transitions were selected for area under the curve based quantitation with MultiQuant 1.2 software (Sciex). For MRM optimization process, the PA14 tn*gidA*::*gidA* strain was used. For QTrap controlling and raw file evaluation the Analyst 1.5.1 software (Sciex) was taken. Samples were measured in biological triplicates. Mean values of transitions and peptides were calculated and used for quantitation. Measured protein abundance was normalized to $^{13}C_6{}^{15}N_2$-L-lysine labeled protein abundance and to the mean of the four housekeeping proteins.

***Relative expression dynamic of gidA ex vivo***

*G. mellonella* larvae were infected with 100 bacteria of the reference strain PA14 tn*ladS* and incubated for 12, 14, 16, or 18 h at 37°C, respectively. The pooled hemolymph of 10 larvae per timepoint was collected and mixed with one volume of RNAprotect, incubated for 10 min at RT and centrifuged at 10.000 x g. RNA from the pellet was extracted using the RNA easy Kit following the manufacturers protocol (Qiagen, Germany). The QuantiFast SYBR Green RT-PCR Kit (Qiagen, Germany) was used with primers for *gidA* and three housekeeping genes (PA14_07700, PA14_55650, PA14_56080) (Table S3) to perform qRT-PCR on a Roche LightCycler 480 device (Roche, Germany). Crossing point (CP) values were calculated using the second derivative maximum method of the LightCycler 480 software. Delta CP-values were calculated as $CP_{gidA}$– $CP_{reference}$ (mean values of housekeeping genes). In total, the experiment was performed with four biological and two technical replicates. *p*-value was calculated using Mann Whitney test.

**Supplementary figures, tables, and datasets**

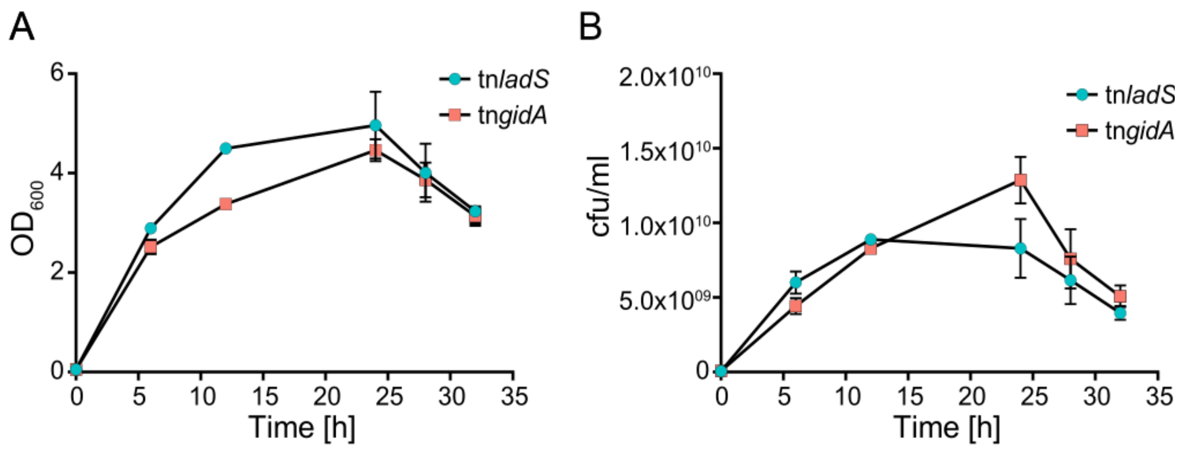


**Figure S1:** Growth of the *P. aeruginosa* PA14 transposon mutant tn*gidA* and the reference tn*ladS* in LB medium was monitored by determining the **(A)** $OD_{600}$ and **(B)** colony forming units (CFU)/ml.

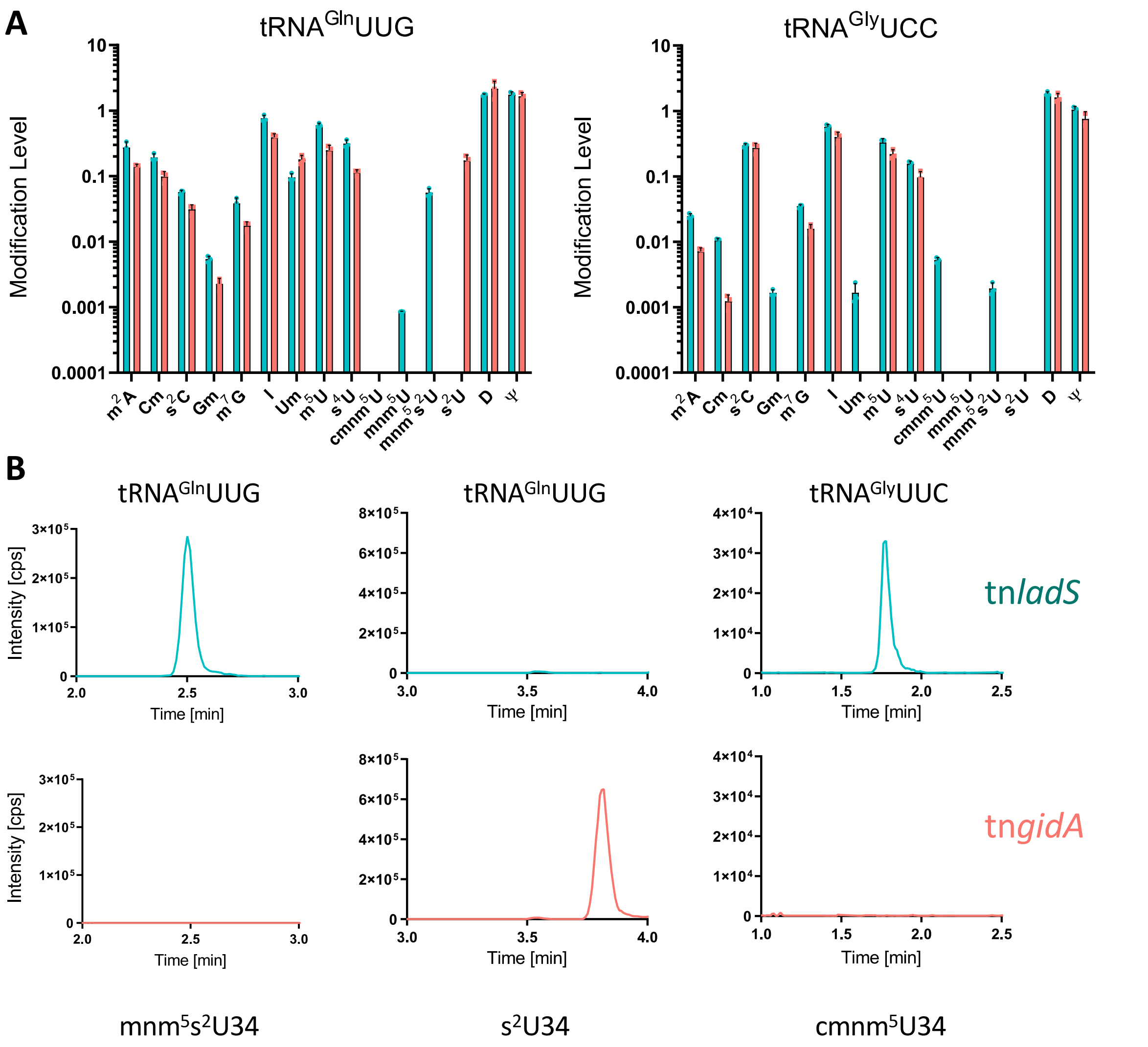


**Figure S2: Quantities of modifications in tRNA$^{Gln}$UUG and tRNA$^{Gly}$UCC from PA14 tn*ladS* and tn*gidA*. (A)** The quantities of modified ribonucleosides for each individual tRNA were analyzed by MRM external standard calibration. The modification level (modified ribonucleoside [fmol] x number of parent ribonucleoside in tRNA sequence / parent ribonucleoside [fmol] + detected modified ribonucleosides that descends from the respective parent nucleosides [fmol]) was determined according Grobe *at al.* 2019 [2]. **(B)** Extracted ion chromatograms (XIC) of identified GidA pathway-related tRNA modifications in tRNA$^{Gln}$UUG and tRNA$^{Gly}$UCC.

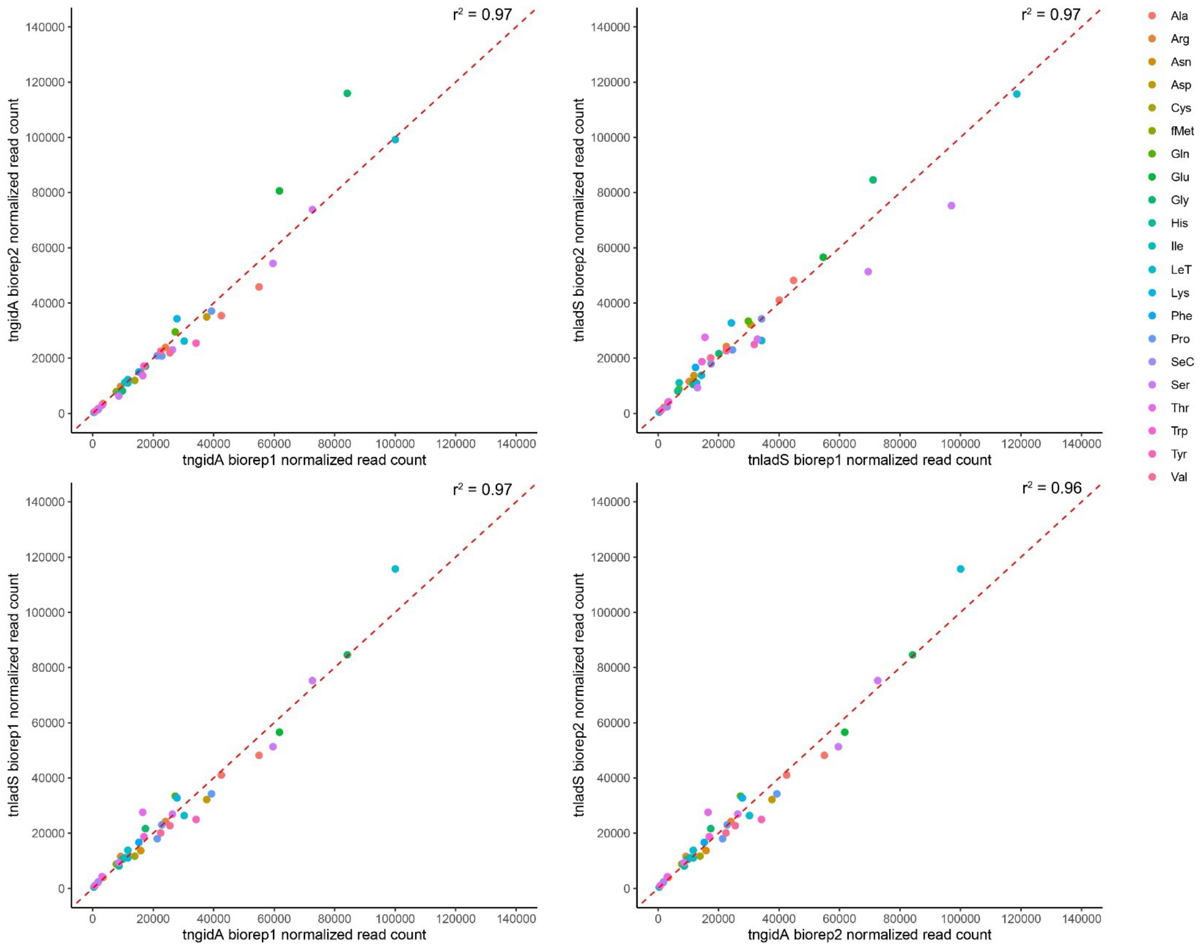


**Figure S3: Quantification of tRNA abundance in tn*gidA* and tn*ladS* strains using Nano-tRNAseq. (A)** Scatter plots of tRNA abundances of each biological replicate of tn*gidA* (left) and tn*ladS* (right). Each color-coded point represents a tRNA alloacceptor (that is the tRNA isoacceptors aminoacylated with identical amino acid). **(B)** Scatter plots of tRNA abundance across biological replicates. The fitted linear regression (red dashed line) and Spearman coefficient of correlation ($r^2$) are indicated for each pair.

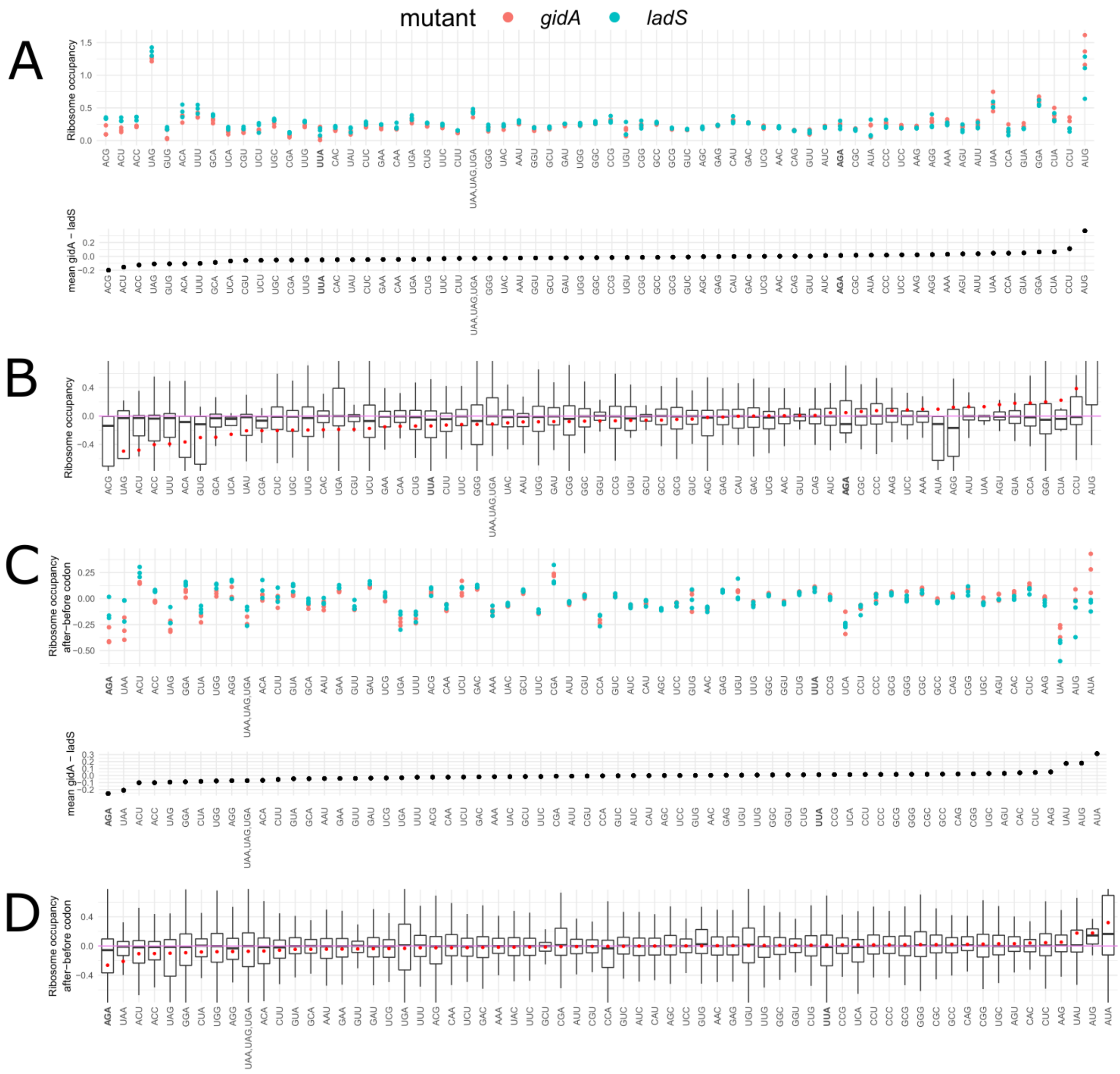


**Figure S4 Mean of the ribosome occupancy for each codon.** The mean of individualized codon's reads (from each biological triplicate of both tn*gidA* and tn*ladS* strains) in each gene was calculated to represent the ribosome occupancy (A site) for each codon throughout the transcriptome. **(A)** Ribosome occupancy at all 64 codons. Mean ribosomal occupancy (y axis) at the certain codons (x axis) for tn*gidA* and tn*ladS* mutants (top) and the mean difference between both mutants (bottom). Codons were sorted based on the mean difference between tn*gidA* and tn*ladS* as shown in the figure at the bottom. **(B)** Boxplots to illustrate the variation in ribosome occupancy at the different codons. Therefore, the mean between the three replicates was calculated for each codon position. The boxplots show the mean difference between tn*gidA* and tn*ladS* whereas the codons are sorted by this mean difference (red dots).**(C)** Mean increase/decrease in ribosome occupancy 9, 12, 15, 18, and 21 nt down- and upstream (y axis) each codon (x axis). The plot at the bottom shows the difference between the mean values of tn*gidA* and tn*ladS*. Codons are sorted according to this mean difference. **(D)** For each difference between the downstream and the upstream regions of the codons (each 9, 12, 15, 18, and 21 nt distance), we calculated the mean of the three replicates. Finally, mean differences between tn*gidA* and tn*ladS* were calculated and shown in boxplots to illustrate the variation of the data. Codons were sorted by the mean values (red dots).

**Table S1: Ion-specific parameters for HPLC-MS/MS-based tRNA modification quantification**. The values for the different parameters were determined after compound optimization measurements with standard analytical compounds at adequate concentrations. Q: Quantifier; I: Identifier; DP: declustering potential; EP: entrance potential; CE: collision energy; CXP: collision cell exit potential; TNV: Tenofovir (IS).

| Analyte | Precursor ion [*m/z*] | Fragment ion [*m/z*] | Dwell Time [ms] | DP [V] | EP [V] | CE [V] | CXP [V] |
|---|---|---|---|---|---|---|---|
| **A Q** | 268.128 | 136.1 | 10 | 61 | 10 | 25 | 12 |
| **A I1** | 268.128 | 119.0 | 10 | 61 | 10 | 63 | 14 |
| **A I2** | 268.128 | 91.9 | 10 | 61 | 10 | 83 | 10 |
| **C Q** | 244.026 | 112.0 | 10 | 36 | 10 | 19 | 10 |
| **C I1** | 244.026 | 95.0 | 10 | 36 | 10 | 57 | 14 |
| **C I2** | 244.026 | 69.0 | 10 | 36 | 10 | 53 | 10 |
| **Cm Q** | 257.995 | 112.0 | 10 | 41 | 10 | 19 | 14 |
| **Cm I1** | 257.995 | 95.0 | 10 | 41 | 10 | 57 | 12 |
| **Cm I2** | 257.995 | 69.1 | 10 | 41 | 10 | 53 | 10 |
| **cmnm$^5$U Q** | 332.072 | 125.0 | 10 | 51 | 10 | 25 | 16 |
| **cmnm$^5$U I1** | 332.072 | 81.9 | 10 | 51 | 10 | 47 | 12 |
| **cmnm$^5$U I2** | 332.072 | 239.1 | 10 | 51 | 10 | 15 | 18 |
| **D Q** | 246.998 | 115 | 10 | 51 | 10 | 13 | 10 |
| **D I1** | 246.998 | 133 | 10 | 51 | 10 | 15 | 12 |
| **D I2** | 246.998 | 211.1 | 10 | 51 | 10 | 17 | 18 |
| **G Q** | 284.061 | 152.1 | 10 | 36 | 10 | 19 | 12 |
| **G I1** | 284.061 | 135.1 | 10 | 36 | 10 | 49 | 12 |
| **G I2** | 284.061 | 110.0 | 10 | 36 | 10 | 49 | 10 |
| **Gm Q** | 298.014 | 152.1 | 10 | 36 | 10 | 23 | 6 |
| **Gm I1** | 298.014 | 135.0 | 10 | 36 | 10 | 55 | 12 |
| **Gm I2** | 298.014 | 109.9 | 10 | 36 | 10 | 55 | 26 |
| **I Q** | 268.900 | 137.0 | 10 | 61 | 10 | 15 | 14 |
| **I I** | 268.900 | 110.1 | 10 | 61 | 10 | 57 | 10 |
| **m$^2$A Q** | 282.237 | 150.1 | 10 | 86 | 10 | 27 | 12 |

| | | | | | | | |
|---|---|---|---|---|---|---|---|
| **$m^2$A I1** | 282.237 | 223.1 | 10 | 86 | 10 | 19 | 16 |
| **$m^2$A I2** | 282.237 | 133.0 | 10 | 86 | 10 | 57 | 12 |
| **$m^5$U Q** | 258.976 | 127.0 | 10 | 36 | 10 | 17 | 12 |
| **$m^5$U I1** | 258.976 | 110.0 | 10 | 36 | 10 | 45 | 10 |
| **$m^7$G Q** | 298.011 | 166.0 | 10 | 31 | 10 | 25 | 22 |
| **$m^7$G I1** | 298.011 | 149.0 | 10 | 31 | 10 | 51 | 12 |
| **$m^7$G I2** | 298.011 | 124.0 | 10 | 31 | 10 | 57 | 10 |
| **$mnm^5s^2$U Q** | 304.102 | 141.1 | 10 | 41 | 10 | 25 | 12 |
| **$mnm^5s^2$U I1** | 304.102 | 81.9 | 10 | 41 | 10 | 45 | 12 |
| **$mnm^5s^2$U I2** | 304.102 | 172.1 | 10 | 41 | 10 | 17 | 14 |
| **$mnm^5$U Q** | 288.039 | 125.1 | 10 | 41 | 10 | 25 | 12 |
| **$mnm^5$U I1** | 288.039 | 156.1 | 10 | 41 | 10 | 17 | 14 |
| **$mnm^5$U I2** | 288.039 | 81.9 | 10 | 41 | 10 | 45 | 12 |
| **$s^2$C Q** | 260.001 | 128.0 | 10 | 41 | 10 | 19 | 10 |
| **$s^2$C I** | 260.001 | 110.9 | 10 | 41 | 10 | 55 | 12 |
| **$s^2$U/$s^4$U Q** | 261.078 | 129.0 | 10 | 41 | 10 | 15 | 14 |
| **$s^2$U/$s^4$U I** | 261.078 | 112.0 | 10 | 41 | 10 | 53 | 10 |
| **TNV Q** | 287.984 | 176.0 | 10 | 60 | 10 | 35 | 14 |
| **TNV I** | 287.984 | 159.0 | 10 | 60 | 10 | 43 | 13 |
| **U Q** | 244.811 | 113.0 | 10 | 51 | 10 | 17 | 10 |
| **U I1** | 244.811 | 70.0 | 10 | 51 | 10 | 51 | 10 |
| **U I2** | 244.811 | 96.0 | 10 | 51 | 10 | 49 | 12 |
| **Um Q** | 259.124 | 113.0 | 10 | 26 | 10 | 15 | 10 |
| **Um I1** | 259.124 | 147.0 | 10 | 26 | 10 | 13 | 12 |
| **Um I2** | 259.124 | 70.0 | 10 | 26 | 10 | 49 | 10 |
| **Ψ Q** | 244.811 | 113.0 | 10 | 51 | 10 | 17 | 10 |
| **Ψ I1** | 244.811 | 70.0 | 10 | 51 | 10 | 51 | 10 |
| **Ψ I2** | 244.811 | 96.0 | 10 | 51 | 10 | 49 | 12 |

Table S2: **Strains and plasmids used in this study.**

| **Strain** | **Description** | **Reference** |
|---|---|---|
| ***Escherichia coli*** | | |
| *E. coli* DH5α | F$^-$ *endA1*, *glnV44*, *thi-1*, *recA1*, *relA1*, *gyrA*96, *deoR*, *nupG*, Φ80*dlacZ*ΔM15, Δ(*lacZYA-argF*)U169, *hsdR17*($r_K^- m_K^+$), λ– | [25] |
| ***Pseudomonas aeruginosa*** | | |
| PA14 tn*ladS* | *ladS* transposon mutant from the NR PA14 transposon mutant library; ID 38371; Gm$^R$ | [26] |
| PA14 tn*gidA* | *gidA* transposon mutant from the NR PA14 transposon mutant library; ID 34284; Gm$^R$ | [26] |
| **Plasmid** | | |
| pME6032 | pVS1-p15A shuttle expression vector; Tc$^R$ | [27] |
| pET21::sfGFP | pET21 with sfGFP sequence | [28] |
| pUCP20 | Shuttle vector, Amp$^R$ | [29] |
| pUCP20::*gidA* | pUCP20 containing the PA14 *gidA* gene cloned into the EcoR1/BamH1 site | This study |
| pME6032::MoR1-*gfp* | pME6032 with Arg motif (AGAAGAAGAAGA) upstream of sfGFP as *Eco*RI/*Bgl*II fragment | This study |
| pME6032::MoR2-*gfp* | pME6032 with Arg motif (AGGAGGAGGAGG) upstream of sfGFP as *Eco*RI/*Bgl*II fragment | This study |
| pME6032::MoG1-*gfp* | pME6032 with Arg motif (GGAGGAGGAGGA) upstream of sfGFP as *Eco*RI/*Bgl*II fragment | This study |
| pME6032::MoG2-*gfp* | pME6032 with Arg motif (GGGGGGGGGGGG) upstream of sfGFP as *Eco*RI/*Bgl*II fragment | This study |
| pME6032::MoL1-*gfp* | pME6032 with Arg motif (TTATTATTATTA) upstream of sfGFP as *Eco*RI/*Bgl*II fragment | This study |
| pME6032::MoL2-*gfp* | pME6032 with Arg motif (TTGTTGTTGTTG) upstream of sfGFP as *Eco*RI/*Bgl*II fragment | This study |
| pME6032::MoRG-*gfp* | pME6032 with Arg motif (AGAGGAAGAGGA) upstream of sfGFP as *Eco*RI/*Bgl*II fragment | This study |
| pME6032::MocRG-*gfp* | pME6032 with Arg motif (CGTGGTCGTGGT) upstream of sfGFP as *Eco*RI/*Bgl*II fragment | This study |
| pME6032::MoRGL-*gfp* | pME6032 with Arg motif (AGAGGATTAAGGGGGTTG) upstream of sfGFP as *Eco*RI/*Bgl*II fragment | This study |

Cm$^R$, chloramphenicol resistant; Gm$^R$, gentamycin resistant; Ap$^R$, ampicillin resistant; Cb$^R$, carbenicillin resistant; Km$^R$, kanamycin resistant; Tc$^R$, tetracycline resistant.

**Table S3: Primers used in this study**

| Primer Name | Primer Sequence |
|---|---|
| **For *gidA* overexpression** | |
| gidA_EcoR1_fw | 5' – CCGGAATTCAGGAGGTGCGTGGTGGATTTCCCTACCCGTTTTG |
| gidA_BamH1_rev | 5' – CGCGGATCCTCAGTGGTGGTGGTGGTGGTGAGCGCTCTGCTCCAGTTG |
| **For Translational efficiency assay** | |
| MoR1 | 5' – CCGGAATTCATGAGAAGAAGAAGAATGAGCAAAGGAGAAGAACTTTTCACTGG |
| MoR2 | 5' – CCGGAATTCATGAGGAGGAGGAGGATGAGCAAAGGAGAAGAACTTTTCACTGG |
| MoG1 | 5' – CCGGAATTCATGGGAGGAGGAGGAATGAGCAAAGGAGAAGAACTTTTCACTGG |
| MoG2 | 5' – CCGGAATTCATGGGGGGGGGGGGGATGAGCAAAGGAGAAGAACTTTTCACTGG |
| MoL1 | 5' – CCGGAATTCATGTTATTATTATTAATGAGCAAAGGAGAAGAACTTTTCACTGG |
| MoL2 | 5' – CCGGAATTCATGTTGTTGTTGTTGATGAGCAAAGGAGAAGAACTTTTCACTGG |
| MoRG | 5' – CCGGAATTCATGAGAGGAAGAGGAATGAGCAAAGGAGAAGAACTTTTCACTGG |
| MocRG | 5' – CCGGAATTCATGCGTGGTCGTGGTATGAGCAAAGGAGAAGAACTTTTCACTGG |
| MoRGL | 5' – CCGGAATTCATGAGAGGATTAAGGGGGTTGATGAGCAAAGGAGAAGAACTTTTCACTGG |
| Motif_BglII_rev | 5' - GAAGATCTTTAGTGGTGGTGGTGGTGGTG |
| **For colony PCR (*in vivo* competition assay, transposon control)** | |
| GidA_check_fw | 5' - AACAGAAAAGCCGCTGCTAA |
| GidA_check_rev | 5' - GTTAAGGATGCGGAACTGGA |
| **For *ex vivo* qRT-PCR** | |
| gidA_fw | 5´ - CGCCGACAAGGAAAGCCAC |
| gidA_rev | 5´ - TGCCACGGATGGAACGCAC |
| PA14_07700_fw | 5´ - GCTGCTGCACTACGACGAAC |
| PA14_07700_rev | 5´ - TCGCGCAATGCTTGCTCC |
| PA14_55650_fw | 5´ - CAGACCGATAGCCTGGAACGAC |
| PA14_55650_rev | 5´ - ATGCCTGCTCCTGCTCGAC |
| PA14_56080_fw | 5´ - CGAGTGGCAAGGCGGCAATAG |
| PA14_56080_rev | 5´ - GAGCATTTCCAGCTTCAGCGAC |
| For purification of individual tRNAs | |
| $tRNA^{Gln}$UUG | 5´-GGCAG GATCA AAACC TGCTG CCTTA CCG - ´3 Biotin |
| $tRNA^{Gly}$UCC | 5´-TCAGC TTGGA AGGCT GAGGC TCTAC CA-´3 Biotin |
| $tRNA^{Arg}$UCU | 5´- ACCAT CCGCT TAGAA GGCGG ATGCT CTATC- ´3 Biotin |

Engineered restriction sites are underlined.

**Dataset: Significantly regulated proteins and their respective PseudoCAP function.** The dataset contains all labeled genes shown in the plot Figure 6A with a log2 fold change between tn*gidA* and tn*ladS* of at least 1.2 (log2FC > 0.263) and a FDR <= 0.05.

**SI References**


1. Hornischer, K., et al., *BACTOME-a reference database to explore the sequence- and gene expression-variation landscape of Pseudomonas aeruginosa clinical isolates.* Nucleic Acids Res, 2018.
2. Grobe, S., et al., *Identification and Quantification of (t)RNA Modifications in Pseudomonas aeruginosa by Liquid Chromatography-Tandem Mass Spectrometry.* Chembiochem, 2019. **20**(11): p. 1430-1437.
3. Su, D., et al., *Quantitative analysis of ribonucleoside modifications in tRNA by HPLC-coupled mass spectrometry.* Nat Protoc, 2014. **9**(4): p. 828-41.
4. Yokogawa, T., et al., *Optimization of the hybridization-based method for purification of thermostable tRNAs in the presence of tetraalkylammonium salts.* Nucleic Acids Res, 2010. **38**(6): p. e89.
5. Essar, D.W., et al., *Identification and characterization of genes for a second anthranilate synthase in Pseudomonas aeruginosa: interchangeability of the two anthranilate synthases and evolutionary implications.* J Bacteriol, 1990. **172**(2): p. 884-900.
6. Koch, A.K., et al., *Hydrocarbon assimilation and biosurfactant production in Pseudomonas aeruginosa mutants.* J Bacteriol, 1991. **173**(13): p. 4212-9.
7. Musken, M., et al., *Genetic determinants of Pseudomonas aeruginosa biofilm establishment.* Microbiology, 2010. **156**(Pt 2): p. 431-41.
8. Musken, M., et al., *A 96-well-plate-based optical method for the quantitative and qualitative evaluation of Pseudomonas aeruginosa biofilm formation and its application to susceptibility testing.* Nat Protoc, 2010. **5**(8): p. 1460-9.
9. Jander, G., L.G. Rahme, and F.M. Ausubel, *Positive correlation between virulence of Pseudomonas aeruginosa mutants in mice and insects.* J Bacteriol, 2000. **182**(13): p. 3843-5.
10. Pawar, V., et al., *In Vivo Efficacy of Antimicrobials against Biofilm-Producing Pseudomonas aeruginosa.* Antimicrob Agents Chemother, 2015. **59**(8): p. 4974-81.
11. Lucas, M.C., et al., *Quantitative analysis of tRNA abundance and modifications by nanopore RNA sequencing.* Nat Biotechnol, 2023.
12. Li, H., et al., *The Sequence Alignment/Map format and SAMtools.* Bioinformatics, 2009. **25**(16): p. 2078-9.
13. Wickham, H., *ggplot2: Elegant Graphics for Data Analysis*. 2016: Springer-Verlag New York.
14. Love, M.I., W. Huber, and S. Anders, *Moderated estimation of fold change and dispersion for RNA-seq data with DESeq2.* Genome Biol, 2014. **15**(12): p. 550.
15. Oh, E., et al., *Selective ribosome profiling reveals the cotranslational chaperone action of trigger factor in vivo.* Cell, 2011. **147**(6): p. 1295-308.
16. Ingolia, N.T., et al., *The ribosome profiling strategy for monitoring translation in vivo by deep sequencing of ribosome-protected mRNA fragments.* Nat Protoc, 2012. **7**(8): p. 1534-50.
17. Engelhardt, F., J. Tomasch, and S. Haussler, *Organism-specific depletion of highly abundant RNA species from bacterial total RNA.* Access Microbiol, 2020. **2**(10): p. acmi000159.
18. Martin, M., *Cutadapt removes adapter sequences from high-throughput sequencing reads.* 2011, 2011. **17**(1): p. 3.
19. Langmead, B. and S.L. Salzberg, *Fast gapped-read alignment with Bowtie 2.* Nat Methods, 2012. **9**(4): p. 357-9.
20. Mohammad, F., et al., *Clarifying the Translational Pausing Landscape in Bacteria by Ribosome Profiling.* Cell Reports, 2016. **14**(4): p. 686-694.
21. Cox, J. and M. Mann, *MaxQuant enables high peptide identification rates, individualized p.p.b.-range mass accuracies and proteome-wide protein quantification.* Nat Biotechnol, 2008. **26**(12): p. 1367-72.
22. Cox, J., et al., *Andromeda: a peptide search engine integrated into the MaxQuant environment.* J Proteome Res, 2011. **10**(4): p. 1794-805.
23. UniProt, C., *Activities at the Universal Protein Resource (UniProt).* Nucleic Acids Res, 2014. **42**(1): p. D191-8.

24. Winsor, G.L., et al., *Pseudomonas Genome Database: improved comparative analysis and population genomics capability for Pseudomonas genomes.* Nucleic Acids Res, 2011. **39**(Database issue): p. D596-600.
25. Woodcock, D.M., et al., *Quantitative evaluation of Escherichia coli host strains for tolerance to cytosine methylation in plasmid and phage recombinants.* Nucleic Acids Res, 1989. **17**(9): p. 3469-78.
26. Liberati, N.T., et al., *An ordered, nonredundant library of Pseudomonas aeruginosa strain PA14 transposon insertion mutants.* Proc Natl Acad Sci U S A, 2006. **103**(8): p. 2833-8.
27. Heeb, S., C. Blumer, and D. Haas, *Regulatory RNA as mediator in GacA/RsmA-dependent global control of exoproduct formation in Pseudomonas fluorescens CHA0.* J Bacteriol, 2002. **184**(4): p. 1046-56.
28. Pedelacq, J.D., et al., *Engineering and characterization of a superfolder green fluorescent protein.* Nat Biotechnol, 2006. **24**(1): p. 79-88.
29. West, S.E., A.K. Sample, and L.J. Runyen-Janecky, *The vfr gene product, required for Pseudomonas aeruginosa exotoxin A and protease production, belongs to the cyclic AMP receptor protein family.* J Bacteriol, 1994. **176**(24): p. 7532-42.
